# A relative analysis to cascaded fractional-order controllers in microgrid non-minimum phase converters using EHO

**DOI:** 10.1038/s41598-025-94690-y

**Published:** 2025-03-25

**Authors:** N. R. Anisha Asmy, J. Ramprabhakar, R. Anand, V. P. Meena, Vinay Kumar Jadoun

**Affiliations:** 1 Department of Electrical and Electronics Engineering, Amrita School of Engineering, Amrita Vishwa Vidyapeetham, Bengaluru, India; 2 Department of Electrical Engineering, National Institute of Technology Jamshedpur, Jamshedpur, Jharkhand 831014 India; 3 Department of Electrical and Electronics Engineering, Manipal Institute of Technology, Manipal Academy of Higher Education, Manipal, Karnataka India

**Keywords:** Fractional-order controller, Non-minimum phase converter, Boost converter, Microgrid, Proportional integral, Renewable energy sources, Photovoltaic system, Energy grids and networks, Electrical and electronic engineering

## Abstract

Microgrids integrate various distributed energy resources to enhance energy reliability and sustainability. Power electronic converters are vital in microgrids since they provide efficient, reliable, and flexible operation. There are numerous controllers available that can be applied to these converters, and lately, fractional-order controllers (FOC) have gathered huge recognition. These controllers provide enhanced flexibility and superior performance in managing dynamic behavior. There are various structures of FOCs, and this article predominantly focuses on comparing different cascaded fractional order controllers (C-FOC). Four distinct topologies of cascaded fractional order proportional integral (C-FOPI) controllers are selected for comparison with one another and with the cascaded proportional integral controller used in a non-minimum phase converter, such as the boost converter employed in a microgrid system. The controllers are optimized using the Elephant Herd Optimization (EHO) algorithm with the Integral of Time-weighted Absolute Error (ITAE) serving as the performance metric. Each controller is subject to variation in system changes, and the outcomes are documented and correlated to ascertain the optimum structure. The simulation outcomes endorsed notable advancements in terms of transient and steady-state performance, featuring improved resilience to parameter changes, a reduction of 36.6% in settling time, 15% in overshoot, 20.1% in rise time, an improved phase margin of more than 51% and more than 50% reduction in performance indices compared to traditional cascaded proportional integral controllers (PI-PI).

## Introduction

The proliferated load demand and the apprehension for climate change have driven the shift towards renewable energy sources (RES) for energy generation. The operating expenses and the contamination of the environment are greatly reduced by replacing systems powered by fossil fuels with RES^1–4^. To improve flexibility and reliability, the energy can be served decentralized by accessing the resources in remote locations^5^. Such a system, which comprises the distributed generation, loads, energy storage devices, security, control, and energy management system, is known as a microgrid^6^. The extensively installed RES in microgrids are wind electric systems and (Photo-voltaic) PV systems^7^. PV arrays require substantial step-up voltages since these power sources often have low-value direct current (DC) outputs^8^. With the advantages of minimal ripples in input current, enhanced dependability, faster response to transients, and better efficiency, the boost converter can be utilized for enhancing the input voltage level to the preferred value^9^. In principle, the DC-DC converter’s modulating control signal can be appropriately adjusted to obtain broad conversion ratios assisted by the controllers^10^.

The uncomplicatedness and ease of deployment make the proportional integral derivative (PID) control strategy dominate more than 90% of industrial controllers^11–13^. These controllers can reduce steady-state errors but may struggle with dynamic response problems, especially in fluctuating load conditions and changes in system parameters, potentially affecting performance in real-world applications^14^. Nevertheless, the supplanting of fixed-order differentiation and integration with the non-integer order one can be done by fractional calculus theory^15^. The fractional-order systems and circuits have evolved greatly and extensively discussed in the literature, which is contributed by major scientists and mathematicians^16^. More precision in depicting physical objects can be attained with fractional calculus when assessed with integer calculus in general^17^. The response of fractional integrals and derivatives is also exemplified with extended memory and components over time scales of varying orders of magnitude^18^. The adaptive system that needs to be regulated and its controller can both be parts of a fractional-order system. Considering the plant model was perhaps originally obtained as a traditional integer order model, in general control practice, only the FOC is more frequently considered^19^. Immense attention has been accorded to the proportional integral-lambda (PI-$\lambda$) controller in fractional-order applications, anchored from the conventional PI algorithm^20^. Moreover, the fractional-order proportional integral (FOPI) controller provides advanced robustness of control due to enhanced capabilities and flexibilities in parameter adjustment compared to traditional conventional integer-order PI controllers^21–25^. In fractional control, the notion of the term flexibility is closely related to the iso-damping property^26^, where a consistent phase margin is accomplished around the unity gain frequency. Thus, sporadic variations in gain nullify the effect on the phase. This aids in stable dynamic response regardless of modeling obscurities and parameter deviations^27^. An enhanced disturbance rejection can also be accomplished with the complementary tuning parameter^28,29^.

Voltage mode control (VMC) with only one control loop is a frequently used technique for the regulation of boost converters in low-power applications. The boost converter has a non-minimum phase nature, and with low values of inductance and capacitance, instability can easily occur due to the transfer function’s negative zero. Whereas to ensure the system’s safety, it is vital to meticulously control the input current. The control structure with an exterior control circuit for voltage and an interior control circuit for current is known by the name ’cascaded control. This type of framework is attaining wide acceptance because of its ability to regulate both voltage and current effectively, respond faster to disturbances, and ensure stability in high-power applications^30^. While cascaded controllers provide feedback with features of local convergence, integers and FOCs still have issues with inferior tracking performance. Therefore, to show its better response, many PI and FOPI cascaded controllers have been examined, and some of the literature is tabulated as shown in Table 1.

**Table 1:** Overview of the cascaded controllers with optimization.

Literature	Controller	Optimization	Key findings	Evaluation Basis
Ali et al.^31^	PI-FOPID	Gorilla Troops Optimizer	Compared to other controllers, the results showed significant enhancement in maximum overshoot, undershoot and settling time	Assessment was under variations in system parameters, load shifts and disturbances and fluctuations in renewable energy sources
Murali et al.^32^	$\hbox {PI-FOPD}^\lambda$	Whale Optimization Algorithm	The sensitivity study demonstrated the effectiveness of the suggested controller	Evaluation in different deregulation environments
Jena et al.^33^	2-DOF-FOPIDN-FOPDN	Wild Goat Algorithm (WGA)	Enhanced dynamic response by reducing settling times and limiting oscillation peaks	Investigation was under the presence of parametric fluctuations and intermittent load perturbations
Khan et al.^34^	$\hbox {PI}^\lambda$(1$+$PDF)	Zebra Optimization Algorithm (ZOA)	Superior performance whilst it exhibits less overshoots, undershoots, and settling times	Evaluation under load variation, performance analysis and sensitivity analysis
Dekaraja et al.^35^	CFOPD-TID	Artificial Flora Algorithm (AFA)	Rapid convergence by the controller diminishes peak variation and settling times	Examination of robustness under diverse uncertain scenarios, such as varying load circumstances, abrupt source disconnection, and abrupt changes in the size of the step load disturbance
Kumar et al.^36^	PID-FOPD	Sine Cosine Algorithm (SCA)	Superior performance in terms of Figure of demerit, settling time and overshoots	Small load perturbations, random load perturbations and sensitivity analysis are used to examine robustness
Jabari et al.^37^	PIDn(1+PD)	Golden Eagle Optimization	As demonstrated results suggested the controller’s potential for robust operation, quick response under dynamic conditions, and minimal error in steady-state	Stability and robustness are tested under a range of perturbations and parameter changes
Jabari et al.^38^	TDn(1+PI)	Bio-dynamic Grasshopper Optimization Algorithm	Performs better than a traditional controller by minimizing overshoot, achieving low ITAE and quicker settling times	Performance across a variety of operating scenarios, including stepwise load adjustments, random load fluctuations, or the presence of nonlinear variables
Jabari et al.^39^	FOPD(1+PI)	Pelican Optimization Algorithm (POA)	Comparative evaluations show that the controllers perform better in terms of robustness, accuracy, and response time	The evaluation was conducted amidst uncertainties in the system and load disturbances
Celik^40^	FOPI-FOPD	Dragonfly Search Algorithm (DSA)	Performance improvement was shown by enhanced settling time, minimal overshoot and undershoot	Investigated when various step load perturbations are present
Celik and Ozturk^41^	F1PD-TI	Salp Swarm Algorithm	By providing the lowest settling time, overshoot and undershoot, it surpasses all of its competitors	The intermittency and volatility of RESs are used to verify the performance

Numerous stochastic optimization methods, including particle swarm optimization (PSO), Nondominated sorting genetic algorithm (NSGA II), chaotic ant swarm (CAS), Sine cosine algorithm (SCA), memorizable-smoothed functional algorithm (MSFA) and Simulated annealing algorithm (SA) have been used commendably to regulate control parameters of the fractional order controller^42^. The presets governing fractional control are tuned in recent research using the Mother optimization algorithm (MOA)^43^, Gorilla troop optimization (GTO)^44^, Grey Wolf optimization (GWO)^45^, Ant-Lion Optimizer^46^, and Gaze cues learning-based Grey Wolf optimizer (GGWO)^47^. Incorporating gaes hues and behavioural complexities of MOA and GTO may result in higher computational demands^48,49^. However, this work employs the EHO algorithm for control parameter tuning in order to benefit from the simplicity of parameter control and implementation compared to CAS and NSGA II which entail intricate processes like chaos theory dynamics or pareto front sorting^50^. Although PSO is regarded to be the least complicated methodology^51^, the clan-based structure of EHO alleviates the potential for premature convergence, which routinely affects PSO^52^.

Despite the fact that several cascaded controllers have been studied in the literature, a comprehensive investigation is lacking that unifies the study of transient response, frequency response, high frequency analysis, performance analysis, efficiency analysis, input fluctuation, step and random load fluctuations, and parameter uncertainties. Existing literature frequently concentrates on discrete elements rather than offering a comprehensive assessment. Nevertheless, it is also typical to investigate the implementation and compare several C-FOPI controller types. A C-FOPI controller that is competent against parameter uncertainty must be designed to offer improved transient and steady-state response for microgrid operation. This study equivalences several C-FOPI controller configurations that are befitted in boost converters for a DC microgrid system. The primary aspects of this research are as follows:

- The assorted forms of C-FOPI controllers have been systematically defined, and the attributes of the controller are calibrated with the EHO algorithm, an advanced metaheuristic technique known for its effective convergence and robustness in complex optimization tasks.
- A comprehensive analysis has been conducted in both temporal and spectral regions to establish the rigor and resilience of the system’s robustness. The system’s accuracy in operation and resilience to disturbances were evaluated by analyzing its steady-state and transient responses in the time domain. To assess the system’s behavior over a range of frequencies and find any potential stability margins or robustness against external perturbations, frequency domain analysis was carried out.
- A thorough investigation has been carried out, encompassing error index evaluation, high frequency response analysis, pole-zero mapping, and efficiency assessment. These analyses provide a deeper insight into the system’s performance, stability and robustness.
- A rigorous evaluation of the system performance dynamic in diverse operational environments has been conducted, including fluctuations in solar irradiation levels, variations in load demand, and alterations to key system parameter changes.

The rest of the manuscript has been organized as outlined: Section "System functionality overview" elucidates the basic structure of the system. The system’s vitality has been critiqued in Section "Stability and robustness analysis". The Section "Simulation and results" provides supporting simulation and discussion of the results. The interpretation of the findings is furnished in Section "Conclusion".

**Fig. 1:**
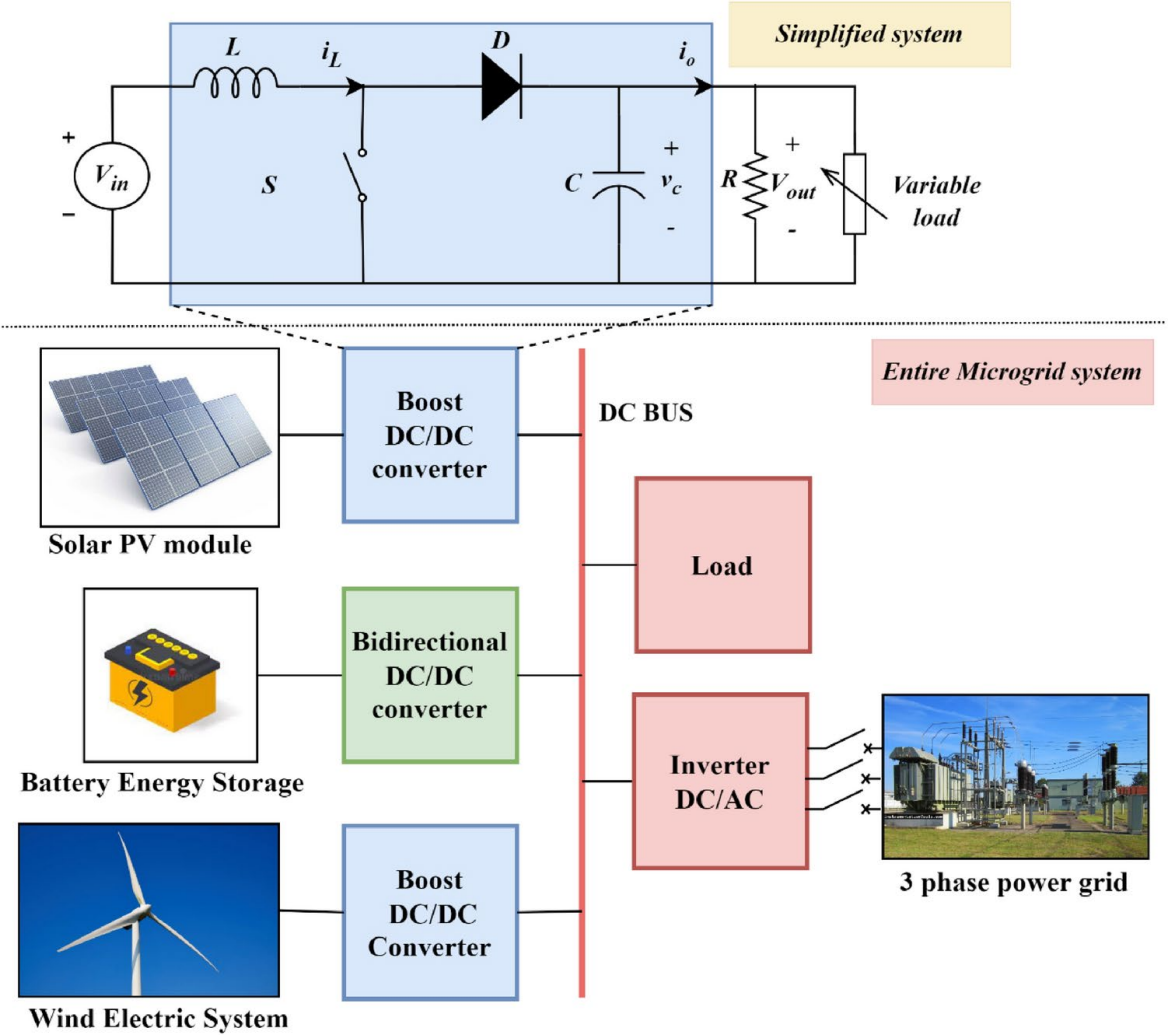
Microgrid system.

## System functionality overview

The Fig. 1 displays the entire microgrid system along with the simplified system that is under consideration. A common DC bus connects the solar PV module, battery energy storage system, and wind electric system. The DC bus can be connected to a local load or to a three-phase power grid through a Direct current / Alternating current (DC/AC) inverter. This manuscript emphasizes the control schemes for a boost converter integrated with a PV system.

### Boost converter

**Fig. 2:**
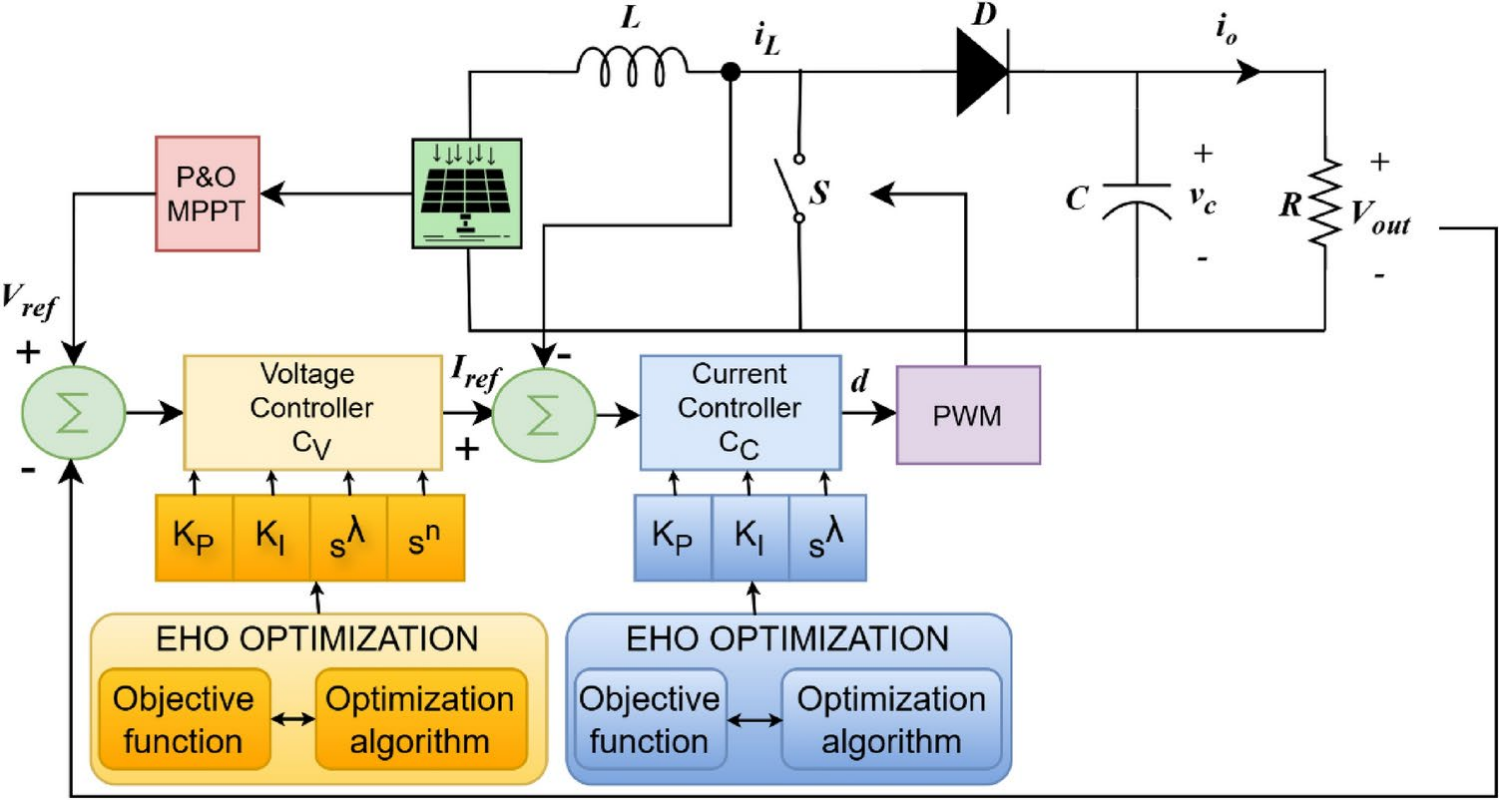
Boost converter system with EHO optimized control structure.

**Fig. 3:**
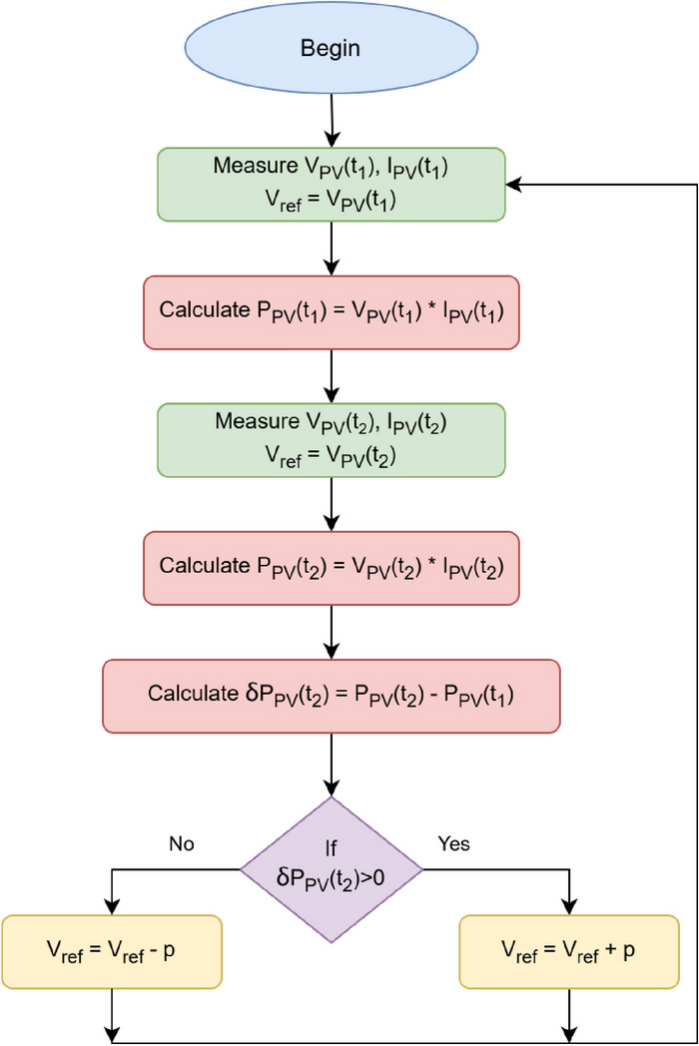
Perturb and observe MPPT algorithm.

Owing to the minimum cost, ingenuous modeling, and least number of components, this paper considers the boost converter, which is widely accepted for the stepping up of voltage for RES at the DC side of the microgrid^53^. Figure 2 portrays the cascaded control technique enforced in the boost converter of the PV system. The $V_{ref}$ is provided by the Perturb and Observe ($P[NONSPACE] \& O$) Maximum power point tracking (MPPT) algorithm. This algorithm is used to record the PV system’s maximum power point due to its effortless application and minimalism^54^. The system’s operational point is varied periodically, and the subsequent change in power $\delta P_{PV}$ is observed intermittently. The algorithm proceeds in the same path with the increase of the power caused by the perturbation *p*; otherwise, the perturbation’s direction is inverted. The system converges to the maximum power point with this iterative approach, as illustrated in Fig. 3. This $V_{ref}$ is compared against the $V_{out}$. The EHO optimized $C_V$ receives the subsequent error and provides the $I_{ref}$. Again, this $I_{ref}$ is compared to the $i_L$. The error is passed to the $C_C$ which likewise optimized through EHO optimization algorithm, resolves the appropriate duty cycle for switching the boost converter’s Insulated-gate bipolar transistor (IGBT). To augment the efficacy of this converter, which is intermittent in time, extremely non-linear, and has a varying structural framework, numerous strategies for modeling have been used. Among the modeling strategies, the transfer function approach is widely used^55^.

**Fig. 4:**
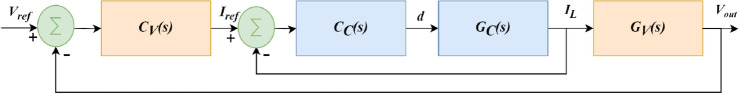
Simplified control diagram of cascaded system.

The simplified cascaded control diagram including the control of both voltage and current is given in Fig. 4.

### Derivation of transfer functions of boost converter from state equations

From the state equations, the loop transfer functions of voltage and current are derived^56^ and detailed as follows:

1 $$\begin{aligned} \begin{bmatrix} \frac{d{i}_L(t)}{dt} \\ \frac{d{v}_{out}(t)}{dt} \end{bmatrix}= & \underbrace{ \begin{bmatrix} 0 & - \frac{(1-d)}{L} \\ \frac{1-d}{C} & -\frac{1}{RC} \end{bmatrix} }_{A} \underbrace{ \begin{bmatrix} i_L \\ v_{out} \end{bmatrix} }_{x} + \underbrace{ \begin{bmatrix} \frac{v_{out}}{L} \\ -\frac{i_L}{C} \end{bmatrix} }_{B_1} d + \underbrace{ \begin{bmatrix} \frac{1}{L} \\ 0 \end{bmatrix} }_{B_2} v_{in} \end{aligned}$$

2 $$\begin{aligned} i_{L}= & \underbrace{ \begin{bmatrix} 1&0 \end{bmatrix} }_{C_1} \begin{bmatrix} i_L \\ v_{out} \end{bmatrix} \end{aligned}$$

3 $$\begin{aligned} v_{out}= & \underbrace{ \begin{bmatrix} 0&1 \end{bmatrix} }_{C_2} \begin{bmatrix} i_L \\ v_{out} \end{bmatrix} \end{aligned}$$

From the state Eqs. (1, 2) and (3), $G_c(s)$ which is the transfer functions derived between the $i_L$ and *d*, and $G_o(s)$ which is the transfer function in the open loop, have been obtained as

4 $$\begin{aligned} G_c(s) = \frac{i_L(s)}{d(s)} = C_1(sI-A)^{-1}B1 \end{aligned}$$

On replacing the coefficients of *A*, $B_1$ and $C_1$, the Eq. (4) can be obtained as

5 $$\begin{aligned} G_c(s) = \begin{bmatrix} 1&0 \end{bmatrix} \left( \begin{bmatrix} s & 0 \\ 0 & s \end{bmatrix} - \begin{bmatrix} 0 & -\frac{(1-d)}{L} \\ \frac{(1-d)}{C} & -\frac{1}{RC} \end{bmatrix} \right) ^{-1} \begin{bmatrix} \frac{v_{out}}{L} \\ -\frac{i_L}{C} \end{bmatrix} \end{aligned}$$

$G_c(s)$ can thus be obtained by rewriting Eq. (5) as shown below

6 $$\begin{aligned} G_c(s) = \frac{i_L(s)}{d(s)} = \frac{2I_L(1-d)+V_{out} C}{LCs^2+\frac{L}{R}s+(1-d)^2} \end{aligned}$$

The voltage transfer function at the outer loop, $G_v(s) = \frac{v_{out}(s)}{i_L(s)}$ is derived as

7 $$\begin{aligned} G_v(s)= & \frac{G_o(s)}{G_c(s)} = \frac{\frac{v_{out}(s)}{d(s)}}{\frac{i_L(s)}{d(s)}} = \frac{v_{out}(s)}{i_L(s)} \end{aligned}$$

8 $$\begin{aligned} G_o(s)= & \frac{v_{out}(s)}{d(s)} = C_2(sI-A)^{-1}B_1 \end{aligned}$$

On substituting the coefficients of *A*, $B_1$ and $C_2$ in Eq. (8), the following equation can be attained

9 $$\begin{aligned} G_o(s) = \begin{bmatrix} 0&1 \end{bmatrix} \left( \begin{bmatrix} s & 0 \\ 0 & s \end{bmatrix} - \begin{bmatrix} 0 & -\frac{(1-d)}{L} \\ \frac{(1-d)}{C} & -\frac{1}{RC} \end{bmatrix} \right) ^{-1} \begin{bmatrix} \frac{v_{out}}{L} \\ -\frac{i_L}{C} \end{bmatrix} \end{aligned}$$

On rewriting Eq. (9), $G_o(s)$ can be derived as follows

10 $$\begin{aligned} G_o(s) = \frac{v_{out}(s)}{d(s)} = \frac{-LI_Ls+V_{out}(1-d)}{LCs^2+\frac{L}{R}s+(1-d)^2} \end{aligned}$$

Thus, with respect to Eq. (7) $G_v(s)$ can be derived as shown below

11 $$\begin{aligned} G_v(s) = \frac{G_o(s)}{G_c(s)} = \frac{v_{out}(s)}{i_L(s)} = \frac{-LI_Ls+V_{out}(1-d)}{V_{out}Cs+2I_L(1-d)} \end{aligned}$$

The boost converter’s parameters are configured to function in continuous conduction mode where the current through the inductor remains positive. As the detailed design has been extensively documented in the literature^57^, it is not reiterated in this study to focus on the novel contribution and avoid redundancy.

PID control-based boost converter works effectively with fluctuations in the load and variations in input voltage^58^. However, it’s unable to countenance disparities in the plant parameters due to the boost converter’s non-minimum phase response^59^. Resilience against plant parameter changes is crucial because temperature rise lowers the electrolytic capacitor’s Equivalent series resistance (ESR) and raises the coil resistance of the inductor as reported in^60^. Furthermore, the working of the boost converter is also restricted by the losses in power resulting from the $r_C$ and $r_L$^61^. In order to address each of these issues, C-FOPI controllers have been articulated.

### Fractional order controllers (FOCs)

The term fractional calculus generalizes the differentiation and integration of the basic operator in non-integer order form. The Eq. (12) represents the operator $_\alpha D_{t}^\delta$ with $\delta$ order derivative or integral^62,63^.

12 $$\begin{aligned} _\alpha D_{t}^\delta f(t) = \left\{ {\begin{array}{*{20}{c}} {\begin{array}{*{20}{c}} {\frac{{{d^\delta }}}{{d{{t}^\delta }}}} f(t)\ \ \ \ \ \ \ \ \ \ \ \ \ \ \ & {\Im \left( \delta \right) > 0} \end{array}}\\ {\begin{array}{*{20}{c}} f(t)\ \ \ \ \ \ \ \ \ \ \ \ \ \ \ \ \ \ \ & {\Im \left( \delta \right) = 0} \end{array}}\\ {\begin{array}{*{20}{c}} {\int \limits _\alpha ^{t} f(t) {{{\left( {d{{\tau }} } \right) }^{ - \delta }}} } \ \ \ \ \ \ \ \ & {\Im \left( \delta \right) < 0} \end{array}} \end{array}} \right. \end{aligned}$$

Within which *t* and $\alpha$ are the upper and lower operation limits. Among the definitions of fractional calculus derivative, Caputo’s definition (13) has been considered in this paper for the limits ($n-1< \delta < n$).

13 $$\begin{aligned} _\alpha D_{t}^\delta f({t}) = \frac{1}{\Gamma (n-\delta )} \int _{\alpha }^{{t}} \frac{f^{(n)}({\tau })}{({t}-{\tau })^{\delta -n+1}}d{\tau }. \end{aligned}$$

There are different forms of FOPI controllers proposed in various literatures^15,64–67^. However, a comparison of the various types of C-FOCs is lacking in the existing literature. The four different cascaded forms of the controllers considered in this article are given in the Eqs. (14)–(18).

#### PI-PI

14 $$\begin{aligned} C_v(s) = K_p + \frac{K_i}{s},\ \ C_c(s) = K_p + \frac{K_i}{s} \end{aligned}$$

#### FOPI-FOPI

15 $$\begin{aligned} C_v(s) = K_p + \frac{K_i}{s^\delta },\ \ C_c(s) = K_p + \frac{K_i}{s^\delta } \end{aligned}$$

#### FO(PI)-FOPI

16 $$\begin{aligned} C_v(s) = K_ps^{1-\delta }+\frac{K_i}{s^\delta }, \ \ C_c(s) = K_p + \frac{K_i}{s^\delta } \end{aligned}$$

#### $\hbox {FO(PI)}^n$-FOPI

17 $$\begin{aligned} C_{v}(s) = \frac{1}{s^n} \left(K_p + \frac{K_i}{s}\right), \ \ C_c(s) = K_p + \frac{K_i}{s^\delta } \end{aligned}$$

#### (FOPI)-FOPI

18 $$\begin{aligned} C_v(s) = K_p\left(1 + \frac{K_i}{s}\right)\frac{1}{s^n}, \ \ C_c(s) = K_p + \frac{K_i}{s^\delta } \end{aligned}$$

where $C_v(s)$ and $C_c(s)$ are the voltage and current controller transfer function. Equation (14) represents the classic cascaded PI controller. The traditional FOPI controllers^15^ are taken into consideration in cascade form by Eq. (15). The fractional orders $\delta$ and *n* are present in distinct ways for the voltage controllers of Eqs. (16)^64^, (17)^67^, and (18)^65^.

The $s^\delta$ and $s^n$, which are the fractional-order operators, cannot be explicitly used in an integer-order system since it is infinite-dimensional. Consequently, it is necessary that these fractional order control systems are approximated by discretization methods. One of the commonly used discretization methods is the Oustaloup method^68^ in which the fractional-order system is approximated by a higher-order continuous integer order system. The parameters for approximation are taken as follows:

- Bandwidth : $$[\omega _a, \omega _b] = [0.001, 10000]$$
- Approximation order : $$N = 4$$

As higher orders of approximation add processing complexity, reducing the effectiveness in real-time implementation, fourth-order approximation was chosen since it can provide the optimal balance between accuracy and computing viability. The dual control strategy for the boost converter is implemented using Eqs. (14)–(18).

### Elephant herd optimization

**Fig. 5:**
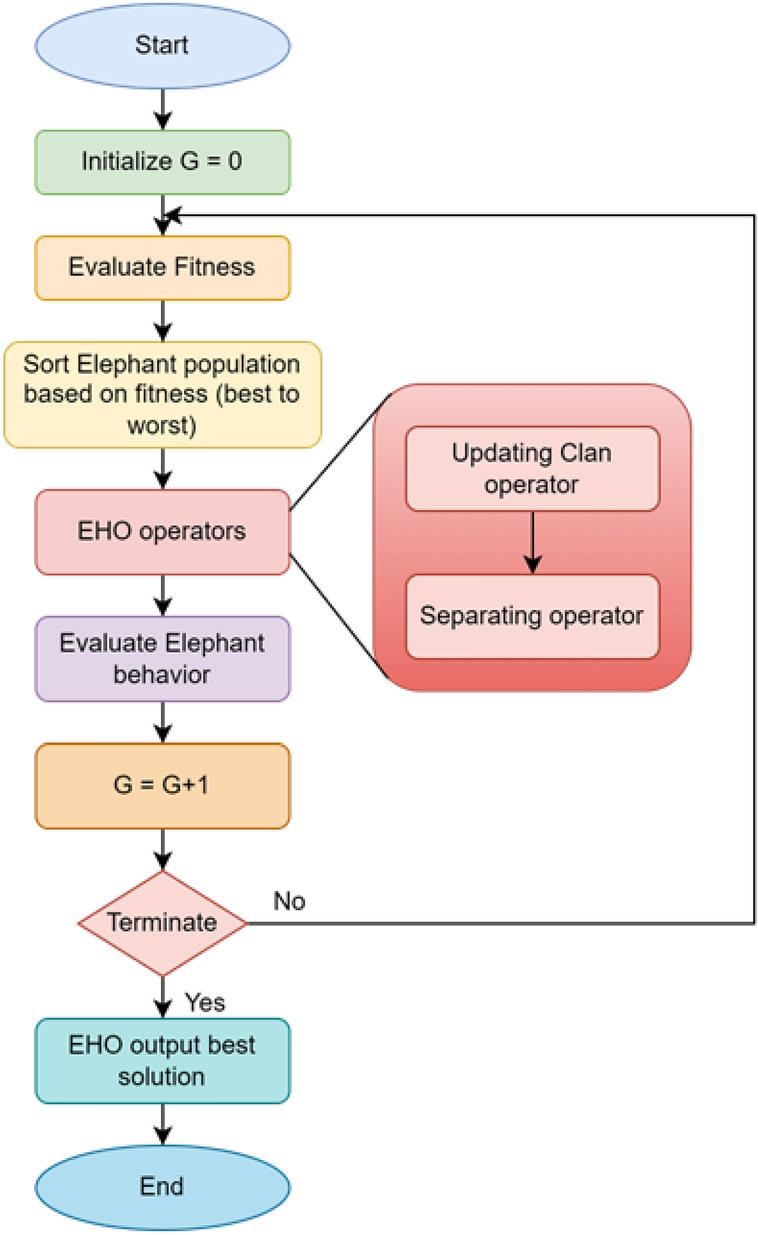
EHO algorithm.

The regulator coefficients $K_p$, $K_i$, $\delta$, and *n* are obtained through EHO algorithm. The use of EHO in this article is due to its less complicated architecture and fewer parameters for control^69^. The EHO takes its cues from how elephant groups herd^70^. Under the guidance of a matriarch, elephants belonging to various clans coexist, and as they mature, they separate from the rest of the group. The clan updating operator and the separating operator are the two operators that result from these characteristics. The clan updating operator adjusts the position of both elephants and the matriarch while the separating operator is put into practice^71^. The EHO algorithm, for which the structural algorithm is shown in Fig. 5, performs exceptionally well in addressing optimization issues, as demonstrated by its applications^72–74^.

**Fig. 6:**
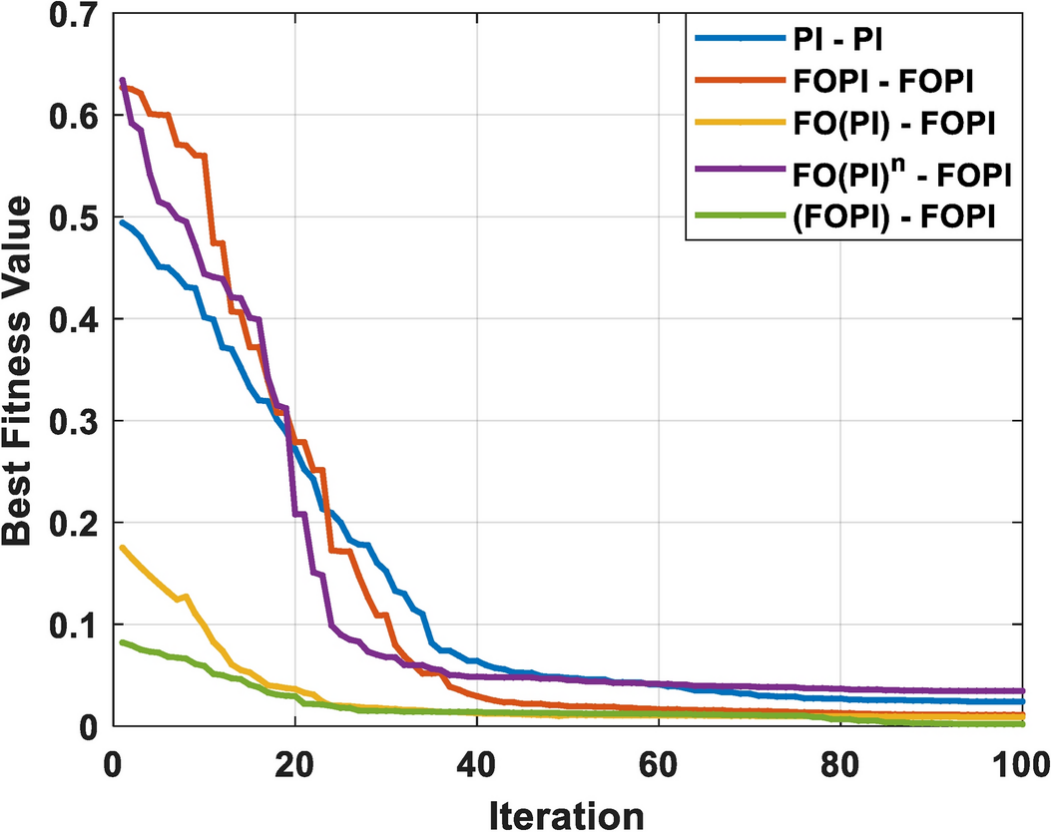
Convergence curve of EHO algorithm.

The execution of the algorithm has been done on 11th Gen Intel(R) Core(TM) i5-1135G7 CPU operating at 1.5 GHz with 8 GB of RAM. The FOC parameters have been tuned with EHO, by which the convergence curve is illustrated in Fig. 6, and the values are shown in Table 2. To enhance the instantaneous response and reduce long term error in control systems, ITAE is particularly pertinent. Since the persistent faults are penalized more severely by weighting the error with time, the system stabilizes rapidly. The following optimization problem is formulated to efficiently adjust the control settings for ITAE.

19 $$\begin{aligned} \begin{aligned} minimize \ ITAE = \int _{0}^{\infty } {t|e(t)|}\ dt \\ in \ accordance \ with \ \ \ \ Kp \ \in {[}K_{p1*},K_{p2*}{]} \\ Ki \ \in {[}K_{i1*},K_{i2*}{]} \\ \delta \ \in {[}0,1{]} \\ {n \ \in {[}0,1{]}} \end{aligned} \end{aligned}$$

The criteria used for this optimization are:

- Number of elephants (population size) = 30
- Number of clans = 5
- Alpha parameter for the clan updating = 0.5
- Beta parameter for the clan updating = 0.1
- Maximum number of iterations = 100
- Dimension of the search space = varies based on the controller structure
- Lower bounds $K_{p1*}$, $K_{i1*}$, $\delta$ and *n* = [0, 0, 0, 0]
- Upper bounds $K_{p2*}, K_{i2*}$, $\delta$ and *n*= [10, 10, 1, 1]

Based on the body of research for controller tuning, the limits of the controller parameters employed in the optimization process were selected^75^. The bounds guarantee adequate tuning freedom for strong transient and steady-state performance while preventing unreasonably high gains that can cause instability. Since the current controller remains unchanged, the gains for voltage controller were individually optimized for each cascaded controller configuration. The optimized $K_{pc}$ and $K_{ic}$ values for PI based current controller are 9.8034 and 7.2238, respectively. For the FOPI—based current controller, the tuned parameters are $K_{pc}$ = 9.7567, $K_{ic}$ = 9.2800, and the fractional order $\delta$ = 0.8690

**Table 2:** Controller parameters as obtained from EHO.

	$\hbox {K}_{{pv}}$	$\hbox {K}_{{iv}}$	$\delta _{v}$	n
PI-PI	0.1282	1.3520	1	–
FOPI-FOPI	6.8841	9.1802	0.0100	–
FO(PI)-FOPI	0.0001	2.9278	0.1320	–
$\hbox {FO(PI)}^n$-FOPI	6.7276	9.9609	1	0.0323
(FOPI)-FOPI	8.6666	2.6175	0.0483	–

The complete transfer function for each controller, incorporating both the EHO controller and system parameters, is presented below:

#### PI-PI

20 $$\begin{aligned} G(s) = \frac{-0.4844 s^4 + 7830 s^3 + 9.039\times 10^4 s^2 + 8.328\times 10^4 s + 1.542\times 10^4}{0.001928 s^5 + 755.5 s^4 + 8774 s^3 + 9.072\times 10^4 s^2 + 8.332\times 10^4 s + 1.542\times 10^4}\ \ \end{aligned}$$

#### FOPI-FOPI

21 $$\begin{aligned} G(s) = \frac{ \begin{aligned}&-2153 s^{38} - 1.374\times 10^7 s^{37} + 3.602\times 10^{11} s^{36} + 4.972\times 10^{15} s^{35} + 2.602\times 10^{19} s^{34} + 7.12\times 10^{22} s^{33} + 1.125\times 10^{26} s^{32} \\&+ 1.076\times 10^{29} s^{31} + 6.382\times 10^{31} s^{30} + 2.386\times 10^{34} s^{29} + 5.681\times 10^{36} s^{28} + 8.693\times 10^{38} s^{27} + 8.617\times 10^{40} s^{26} \\&+ 5.566\times 10^{42} s^{25} + 2.356\times 10^{44} s^{24} + 6.572\times 10^{45} s^{23} + 1.212\times 10^{47} s^{22} + 1.48\times 10^{48} s^{21} + 1.195\times 10^{49} s^{20}\\&+ 6.365\times 10^{49} s^{19} + 2.238\times 10^{50} s^{18} + 5.186\times 10^{50} s^{17} + 7.918\times 10^{50} s^{16} + 8.001\times 10^{50} s^{15} + 5.384\times 10^{50} s^{14} \\&+ 2.425\times 10^{50} s^{13} + 7.335\times 10^{49} s^{12} + 1.491\times 10^{49} s^{11} + 2.031\times 10^{48} s^{10} + 1.84\times 10^{47} s^9 + 1.095\times 10^{46} s^8 \\&+ 4.224\times 10^{44} s^7+ 1.045\times 10^{43} s^6 + 1.635\times 10^{41} s^5 + 1.584\times 10^{39} s^4 + 9.219\times 10^{36} s^3 + 3.077\times 10^{34} s^2 \\&+ 5.356\times 10^{31} s + 3.737\times 10^{28} \end{aligned} }{ \begin{aligned}&5.876 s^{39} + 3.471\times 10^6 s^{38} + 7.635\times 10^{10} s^{37} + 6.623\times 10^{14} s^{36} + 2.952\times 10^{18} s^{35} + 7.468\times 10^{21} s^{34} + 1.127\times 10^{25} s^{33} \\&+ 1.044\times 10^{28} s^{32} + 6.041\times 10^{30} s^{31} + 2.208\times 10^{33} s^{30} + 5.138\times 10^{35} s^{29} + 7.683\times 10^{37} s^{28} + 7.453\times 10^{39} s^{27} \\&+ 4.749\times 10^{41} s^{26} + 2.023\times 10^{43} s^{25} + 5.899\times 10^{44} s^{24} + 1.206\times 10^{46} s^{23} + 1.76\times 10^{47} s^{22} + 1.832\times 10^{48} s^{21}\\&+ 1.341\times 10^{49} s^{20} + 6.765\times 10^{49} s^{19} + 2.31\times 10^{50} s^{18} + 5.272\times 10^{50} s^{17} + 7.988\times 10^{50} s^{16} + 8.04\times 10^{50} s^{15}\\&+ 5.399\times 10^{50} s^{14} + 2.429\times 10^{50} s^{13} + 7.343\times 10^{49} s^{12} + 1.492\times 10^{49} s^{11} + 2.032\times 10^{48} s^{10} + 1.84\times 10^{47} s^9 \\&+ 1.095\times 10^{46} s^8 + 4.225\times 10^{44} s^7 + 1.045\times 10^{43} s^6 + 1.635\times 10^{41} s^5 + 1.584\times 10^{39} s^4 + 9.219\times 10^{36} s^3 \\&+ 3.077\times 10^{34} s^2 + 5.356\times 10^{31} s + 3.737\times 10^{28} \end{aligned} } \end{aligned}$$

#### FO(PI)-FOPI

22 $$\begin{aligned} G(s) = \frac{ \begin{aligned}&-1.15 s^{30} - 4586 s^{29} + 2.043\times 10^8 s^{28} + 2.17\times 10^{12} s^{27} + 8.53\times 10^{15} s^{26} + 1.577\times 10^{19} s^{25} + 1.502\times 10^{22} s^{24} \\&+ 7.673\times 10^{24} s^{23} + 2.124\times 10^{27} s^{22} + 3.252\times 10^{29} s^{21} + 2.8\times 10^{31} s^{20} + 1.37\times 10^{33} s^{19} + 3.898\times 10^{34} s^{18} \\&+ 6.604\times 10^{35} s^{17} + 6.734\times 10^{36} s^{16} + 4.135\times 10^{37} s^{15} + 1.519\times 10^{38} s^{14} + 3.306\times 10^{38} s^{13} + 4.219\times 10^{38} s^{12} \\&+ 3.18\times 10^{38} s^{11} + 1.434\times 10^{38} s^{10} + 3.893\times 10^{37} s^9 + 6.348\times 10^{36} s^8 + 6.148\times 10^{35} s^7 + 3.461\times 10^{34} s^6 \\&+ 1.097\times 10^{33} s^5 + 1.922\times 10^{31} s^4 + 1.811\times 10^{29} s^3 + 8.668\times 10^{26} s^2 + 1.947\times 10^{24} s + 1.626\times 10^{21} \end{aligned} }{ \begin{aligned}&4.726 s^{30} + 3.408\times 10^6 s^{29} + 4.169\times 10^{10} s^{28} + 1.905\times 10^{14} s^{27} + 4.079\times 10^{17} s^{26} + 4.431\times 10^{20} s^{25} + 2.576\times 10^{23} s^{24}\\&+ 8.188\times 10^{25} s^{23} + 1.443\times 10^{28} s^{22} + 1.447\times 10^{30} s^{21} + 8.421\times 10^{31} s^{20} + 2.917\times 10^{33} s^{19} + 6.266\times 10^{34} s^{18}\\&+ 8.622\times 10^{35} s^{17} + 7.697\times 10^{36} s^{16} + 4.397\times 10^{37} s^{15} + 1.561\times 10^{38} s^{14} + 3.346\times 10^{38} s^{13} + 4.242\times 10^{38} s^{12}\\&+ 3.188\times 10^{38} s^{11} + 1.436\times 10^{38} s^{10} + 3.896\times 10^{37} s^9 + 6.351\times 10^{36} s^8 + 6.149\times 10^{35} s^7 + 3.461\times 10^{34} s^6 \\&+ 1.098\times 10^{33} s^5 + 1.922\times 10^{31} s^4 + 1.811\times 10^{29} s^3 + 8.668\times 10^{26} s^2 + 1.947\times 10^{24} s + 1.626\times 10^{21} \end{aligned} } \end{aligned}$$

#### $\hbox {FO(PI)}^n$-FOPI

23 $$\begin{aligned} G(s) = \frac{ \begin{aligned}&-1.15 s^{30} + 1446 s^{29} + 1.799\times 10^8 s^{28} + 1.313\times 10^{12} s^{27} + 3.918\times 10^{15} s^{26}+ 5.569\times 10^{18} s^{25} + 4.142\times 10^{21} s^{24} \\&+ 1.665\times 10^{24} s^{23} + 3.617\times 10^{26} s^{22} + 4.384\times 10^{28} s^{21} + 3.012\times 10^{30} s^{20} + 1.178\times 10^{32} s^{19} + 2.711\times 10^{33} s^{18} \\&+ 3.745\times 10^{34} s^{17} + 3.117\times 10^{35} s^{16} + 1.554\times 10^{36} s^{15} + 4.627\times 10^{36} s^{14} + 8.145\times 10^{36} s^{13} + 8.375\times 10^{36} s^{12} \\&+ 5.12\times 10^{36} s^{11} + 1.885\times 10^{36} s^{10} + 4.175\times 10^{35} s^{9} + 5.524\times 10^{34} s^{8} + 4.323\times 10^{33} s^{7} + 1.953\times 10^{32} s^{6} \\&+ 4.912\times 10^{30} s^{5} + 6.835\times 10^{28} s^{4} + 5.122\times 10^{26} s^{3} + 1.939\times 10^{24} s^{2} + 3.511\times 10^{21} s + 2.426\times 10^{18} \end{aligned} }{ \begin{aligned}&0.00346 s^{31} + 2017 s^{30} + 2.944\times 10^7 s^{29} + 1.655\times 10^{11} s^{28} + 4.452\times 10^{14} s^{27} + 6.025\times 10^{17} s^{26} + 4.358\times 10^{20} s^{25}\\&+ 1.721\times 10^{23} s^{24} + 3.693\times 10^{25} s^{23} + 4.442\times 10^{27} s^{22} + 3.049\times 10^{29} s^{21} + 1.211\times 10^{31} s^{20} + 2.942\times 10^{32} s^{19} \\&+ 4.621\times 10^{33} s^{18} + 4.89\times 10^{34} s^{17} + 3.508\times 10^{35} s^{16} + 1.631\times 10^{36} s^{15} + 4.716\times 10^{36} s^{14} + 8.208\times 10^{36} s^{13} \\&+ 8.403\times 10^{36} s^{12} + 5.128\times 10^{36} s^{11} + 1.886\times 10^{36} s^{10} + 4.177\times 10^{35} s^{9} + 5.525\times 10^{34} s^{8} + 4.323\times 10^{33} s^{7} \\&+ 1.953\times 10^{32} s^{6} + 4.912\times 10^{30} s^{5} + 6.835\times 10^{28} s^{4} + 5.122\times 10^{26} s^{3} + 1.939\times 10^{24} s^{2} + 3.511\times 10^{21} s + 2.426\times 10^{18} \end{aligned} } \end{aligned}$$

#### (FOPI)-FOPI

24 $$\begin{aligned} G(s) = \frac{ \begin{aligned}&-3.377 s^{38} - 1.284\times 10^4 s^{37} + 5.378\times 10^8 s^{36} + 6.544\times 10^{12} s^{35} + 3.293\times 10^{16} s^{34} + 8.875\times 10^{19} s^{33}\\&+ 1.381\times 10^{23} s^{32} + 1.303\times 10^{26} s^{31} + 7.647\times 10^{28} s^{30} + 2.814\times 10^{31} s^{29} + 6.51\times 10^{33} s^{28}+ 9.558\times 10^{35} s^{27} \\&+ 8.964\times 10^{37} s^{26} + 5.363\times 10^{39} s^{25} + 2.039\times 10^{41} s^{24} + 4.96\times 10^{42} s^{23} + 7.749\times 10^{43} s^{22} + 7.763\times 10^{44} s^{21}\\&+ 4.973\times 10^{45} s^{20} + 2.052\times 10^{46} s^{19} + 5.489\times 10^{46} s^{18} + 9.546\times 10^{46} s^{17} + 1.082\times 10^{47} s^{16} + 8.084\times 10^{46} s^{15}\\&+ 4.025\times 10^{46} s^{14} + 1.342\times 10^{46} s^{13} + 2.999\times 10^{45} s^{12} + 4.479\times 10^{44} s^{11} + 4.456\times 10^{43} s^{10} + 2.931\times 10^{42} s^9\\&+ 1.258\times 10^{41} s^8 + 3.469\times 10^{39} s^7 + 6.113\times 10^{37} s^6 + 6.816\times 10^{35} s^5 + 4.707\times 10^{33} s^4 + 1.948\times 10^{31} s^3 \\&+ 4.66\times 10^{28} s^2 + 5.923\times 10^{25} s + 3.095\times 10^{22} \end{aligned} }{ \begin{aligned}&0.005001 s^{39} + 2939 s^{38} + 5.702\times 10^7 s^{37} + 4.606\times 10^{11} s^{36} + 1.982\times 10^{15} s^{35} + 4.929\times 10^{18} s^{34} + 7.337\times 10^{21} s^{33} \\&+ 6.749\times 10^{24} s^{32} + 3.914\times 10^{27} s^{31} + 1.442\times 10^{30} s^{30} + 3.389\times 10^{32} s^{29} + 5.149\times 10^{34} s^{28} + 5.121\times 10^{36} s^{27} \\&+ 3.36\times 10^{38} s^{26} + 1.463\times 10^{40} s^{25} + 4.275\times 10^{41} s^{24} + 8.429\times 10^{42} s^{23} + 1.121\times 10^{44} s^{22} + 9.986\times 10^{44} s^{21}\\&+ 5.898\times 10^{45} s^{20} + 2.304\times 10^{46} s^{19} + 5.943\times 10^{46} s^{18} + 1.009\times 10^{47} s^{17} + 1.126\times 10^{47} s^{16} + 8.328\times 10^{46} s^{15} \\&+ 4.119\times 10^{46} s^{14} + 1.368\times 10^{46} s^{13} + 3.047\times 10^{45} s^{12} + 4.54\times 10^{44} s^{11} + 4.511\times 10^{43} s^{10} + 2.964\times 10^{42} s^9\\&+ 1.271\times 10^{41} s^8 + 3.504\times 10^{39} s^7 + 6.172\times 10^{37} s^6 + 6.88\times 10^{35} s^5 + 4.75\times 10^{33} s^4 + 1.966\times 10^{31} s^3 \\&+ 4.701\times 10^{28} s^2 + 5.975\times 10^{25} s + 3.121\times 10^{22} \end{aligned} } \end{aligned}$$

## Stability and robustness analysis

The system’s time and frequency response characteristics have been assessed using the transfer function for all the controllers, and the respective metrics can be found in Table 3. The table below shows the results of tuning the system controllers using PSO as well, for an equitable comparison with EHO.

**Table 3:** Response data in time and frequency domain.

	Rise time (s)	Peak overshoot (%)	Settling time (s)	Steady state error	Phase margin (deg)	Gain margin (dB)
EHO PI-PI	0.0914	28.7	0.7156	0.001	52.545	63.8655
EHO FOPI-FOPI	0.0738	25.97	0.4537	− 0.006	53.0076	63.8138
EHO FO(PI)-FOPI	0.0730	13.9214	0.4530	− 0.0005	79.4	14.2
EHO $\hbox {FO(PI)}^n$-FOPI	0.0922	29.719	0.7252	− 0.0066	51.144	64.6618
EHO (FOPI)-FOPI	0.0921	0.2693	0.1531	0.0063	88.1336	58.5
PSO PI-PI	0.337	12.6	2.08	− 0.001	75.1936	71.6203
PSO FOPI-FOPI	0.3	9.81	1.62	− 0.001	74.2889	71.61
PSO FO(PI)-FOPI	0.36	4.46	2.16	− 0.002	100.9232	21.2
PSO $\hbox {FO(PI)}^n$-FOPI	0.374	67	8.54	− 0.0035	17.5379	115
PSO (FOPI)-FOPI	0.591	0	–	0.002	92.2549	71.62

**Fig. 7:**
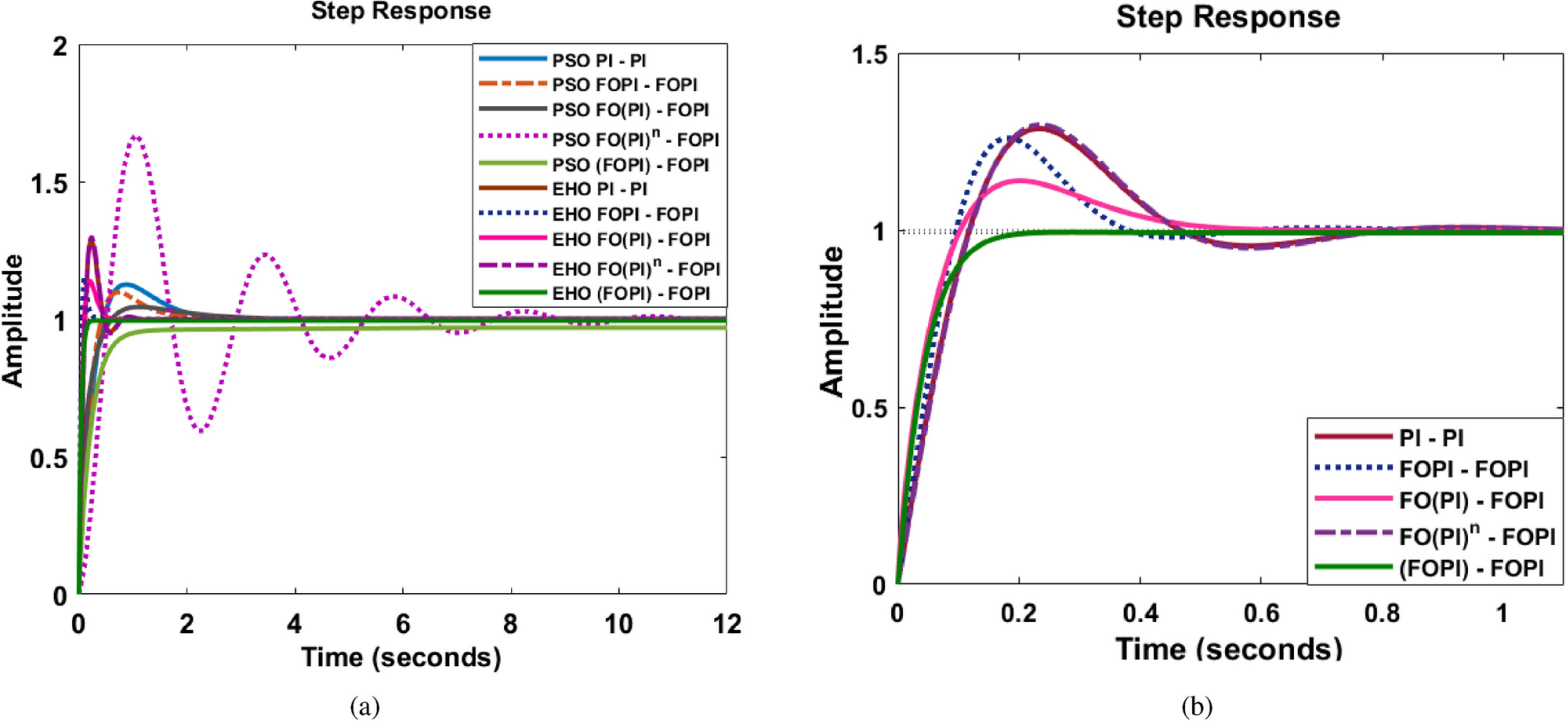
Time response of the system with controllers (**a**) PSO tuned controllers and EHO tuned controllers (**b**) EHO tuned controllers.

### Inference from transient and frequency response

**Fig. 8:**
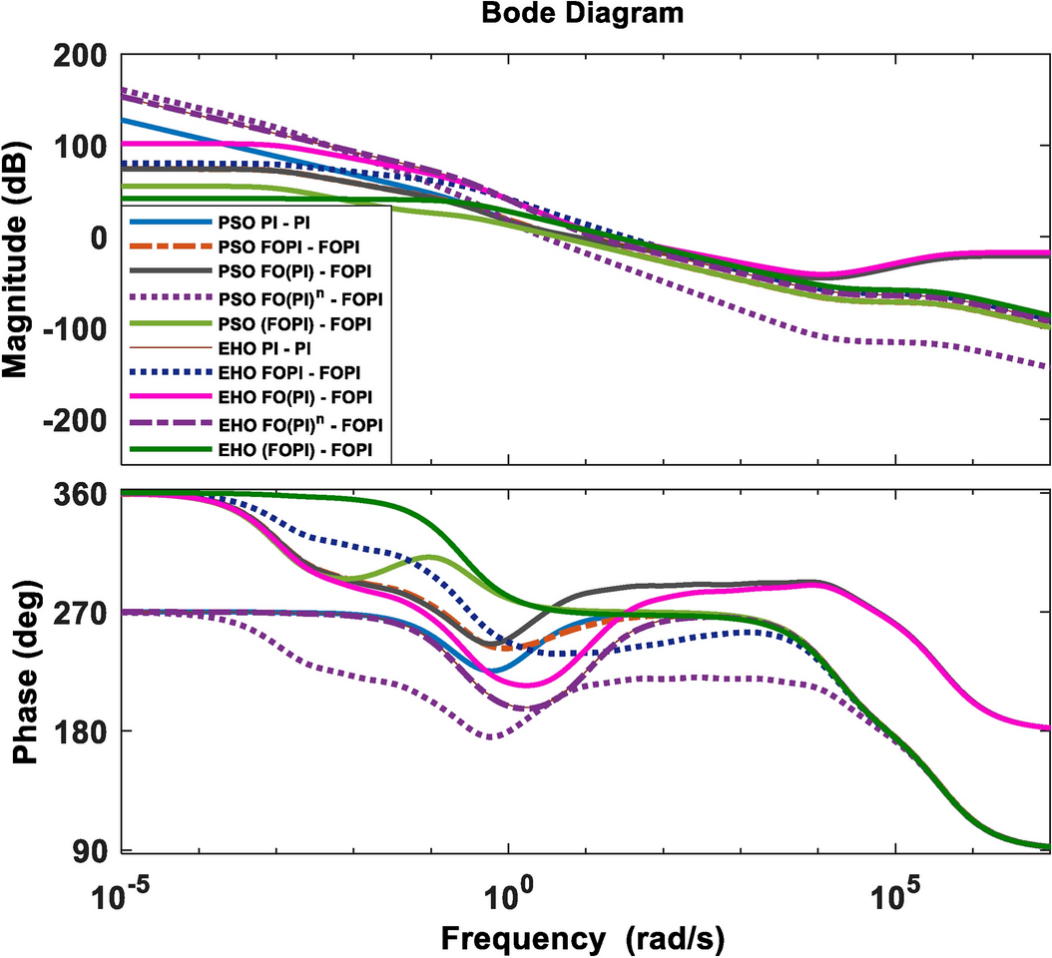
Frequency response of the system with the controllers PSO tuned controllers and EHO tuned controllers.

**Fig. 9:**
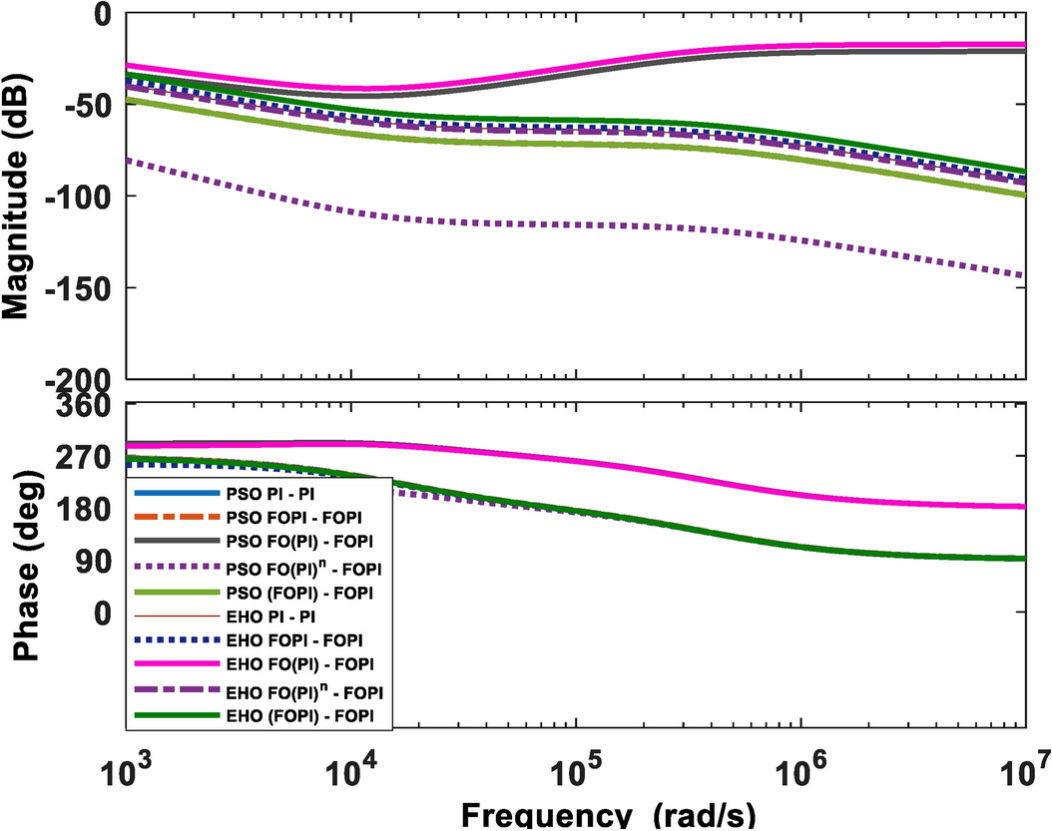
Frequency response of the system magnified at high frequencies.

A phase margin of at least 75 degrees is necessary to achieve an improved output voltage response with minimal overshoot^76^. However, a larger gain and phase margin result in a very stable feedback system but with a very sluggish response. In addition, for the system to be resistant to parameter fluctuation, at the frequency of gain crossover, the phase profile must maintain stability. In majority of the evaluated parameters, EHO tuned controllers exhibit enhanced performance over their PSO tuned counterparts. Comparatively, the EHO FO(PI)-FOPI controller provides a better response in all other parameters except settling time and peak overshoot, as perceived in Figs. 7, 8, 9 and Table 3. This controller thus demonstrates a superior transient response, exhibiting a well-balanced trade-off between overshoot, settling time, and steady-state accuracy. On the other hand, the EHO (FOPI)-FOPI appears overdamped, which, while contributing to stability, may lead to a sluggish response that is not optimal for dynamic load conditions. The optimal selection and placement of $\lambda _c$ and $\lambda _v$ increases the system robustness in this controller.

### High-frequency behaviour analysis

At high frequencies, the magnitude response should delay sharply to avoid noise amplification. A magnified plot of bode at high frequencies is illustrated in Fig. 9. A slower gain decay rate for the EHO FO(PI)–FOPI and PSO FO(PI)–FOPI controller increases the stability against uncertainties, but it may be more susceptible to high-frequency noise. In contrast, the other controllers suppress high-frequency disturbances more effectively than EHO FO(PI)–FOPI and PSO FO(PI)–FOPI controllers because they exhibit a steeper gain roll-off and higher noise rejection.

### Inference from performance indices

An analysis of the performance indices for each controller observed from Table 4 indicates that although the EHO (FOPI)-FOPI controller provides significantly reduced error for all metrics, a substantial steady-state inaccuracy can be seen in time response from Table 3. The second-best metric values of minimum error are provided by the EHO FO(PI)-FOPI controller. The ITAE metric meritoriously validated the enhanced transient response and steady-state accuracy of all the controllers, as illustrated in Fig. 7 and Table 3.

**Table 4:** Performance indices data.

	IAE	ISE	ITAE	ITSE	$\hbox {IT}^2$ AE	$\hbox {IT}^2$ SE
EHO PI-PI	0.1221	0.0473	0.0242	0.0043	0.0092	0.001
EHO FOPI-FOPI	0.088	0.0371	0.0112	0.0022	0.0026	3.64 × 10^−4^
EHO FO(PI)-FOPI	0.0667	0.0228	0.0091	0.0011	0.0024	1.97 × 10^−4^
EHO $\hbox {FO(PI)}^n$-FOPI	0.148	0.0581	0.0346	0.0065	0.0153	0.0018
EHO (FOPI)-FOPI	0.0447	0.0227	0.002	4.95 × 10^−4^	1.87 × 10^−4^	2.16 × 10^−5^
PSO PI-PI	0.3005	0.1127	0.1761	0.0208	0.2144	0.0152
PSO FOPI-FOPI	0.237	0.0983	0.1035	0.0119	0.1092	5.48 × 10^−3^
PSO FO(PI)-FOPI	0.2029	0.0667	0.1181	0.0080	0.1802	5.15 × 10^−3^
PSO $\hbox {FO(PI)}^n$-FOPI	1.5424	0.6396	3.4729	0.7519	14.6961	1.7681
PSO (FOPI)-FOPI	0.2911	0.1242	0.1194	1.83 × 10^−2^	1.11 × 10^−1^	7.82 × 10^−3^

**Fig. 10:**
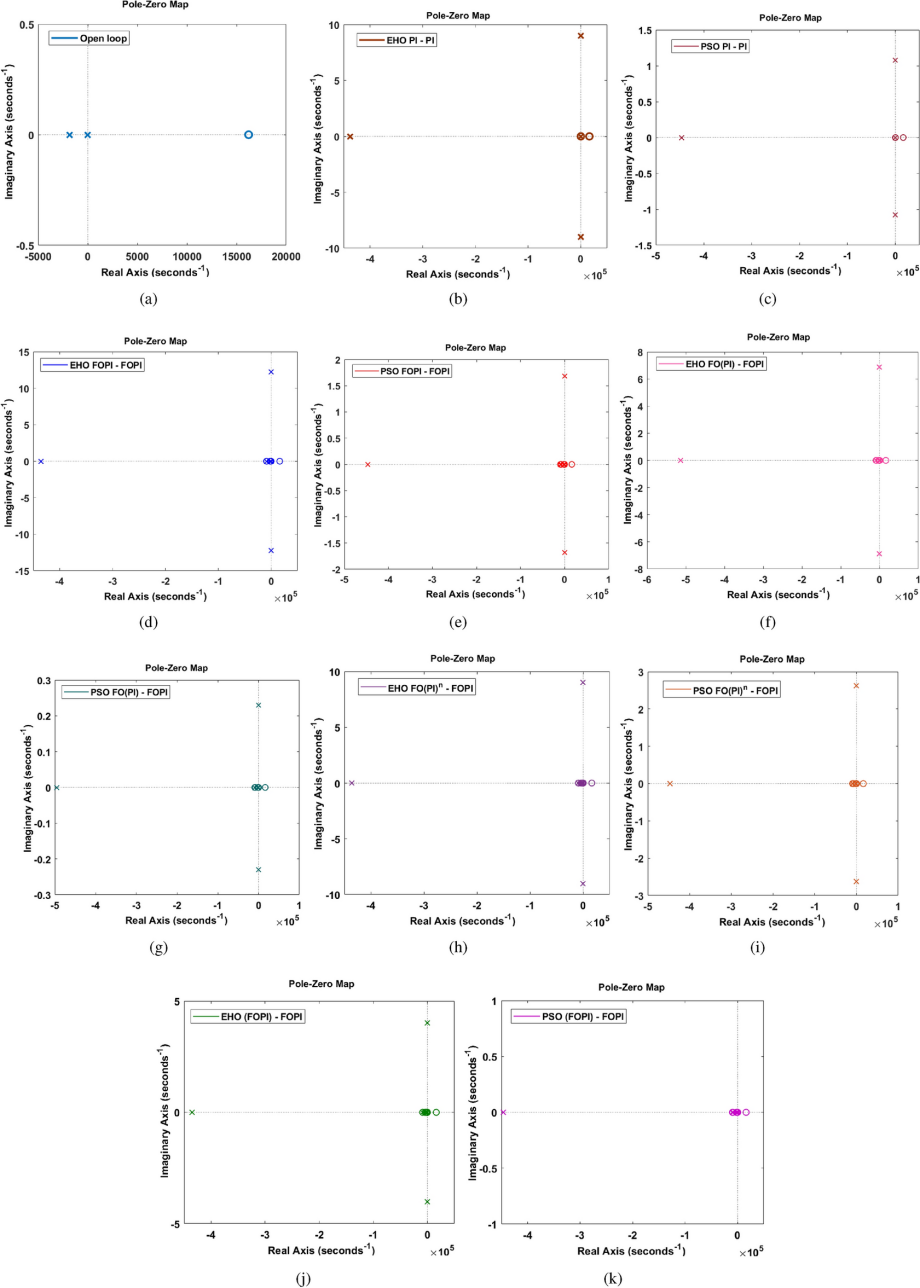
Pole-zero map for (**a**) open loop system without controllers (**b**) with EHO PI-PI controller (**c**) with PSO PI-PI controller (**d**) with EHO FOPI-FOPI controller (**e**) with PSO FOPI-FOPI controller (**f**) with EHO FO(PI)-FOPI controller (**g**) with PSO FO(PI)-FOPI controller (**h**) with EHO $\hbox {FO(PI)}^n$-FOPI controller (**i**) with PSO $\hbox {FO(PI)}^n$-FOPI controller (**j**) with EHO (FOPI)-FOPI controller (**k**) with PSO (FOPI)-FOPI controller.

### Inference from pole-zero analysis

The dynamic behaviour of the system under various control strategies are depicted by the pole-zero maps shown in Fig. 10. An unstable or poorly damped system is indicated by the presence of system poles with a dominating right-half plane zero component, as illustrated by the open-loop pole-zero plot in Fig. 10a. Without control intervention, the presence of the pole on the zero of the real axis raises the possibility of instability. In contrast to the open-loop scenario, the poles are moved farther into the left half plane when a traditional PI controller is used. On the other hand, oscillatory activity with relatively little damping is indicated by the clustering of poles close to the imaginary axis in PSO-tuned controllers.

Significant changes in pole placements result from the addition of the EHO-tuned FOPI controllers, illustrating their influence on the transient response and system stability. The Pole-zero map of different C-FOPI controllers tuned with EHO and PSO implemented with the system is presented in Fig. 10d–k, each of which has a unique effect on the closed-loop dynamics. When compared to the PI controller, the poles are moved farther into the left half plane by the EHO-tuned FO(PI)-FOPI controller with better damping, suggesting increased system stability.

## Simulation and results

The MATLAB/Simulink environment has been utilized to simulate the microgrid environment. A solar PV module and the boost converter parameters considered for simulation are tabulated in Table 5. To accord with the DC/AC inverters, the amount of resistive load is determined for the converter system with 600 V voltage at the terminal. Since high voltage in a reduced power system necessitates rigorous control techniques to provide reliable performance under fluctuating loads, a low output power of 214 W is maintained. This will demonstrate the resilience of the controllers. According to the earlier analysis, which showed that EHO-tuned controllers performed better than PSO-tuned controllers, future simulation results will only consider EHO-tuned controllers.

**Table 5:** Solar PV module and Boost converter parameters considered for simulation.

Solar PV module	
Maximum output voltage $V_{max}$	37.92 V
Open circuit voltage of a single cell $V_{ocs}$	3 V
Voltage at open circuit $V_{oc}$	46.10 V
Current at short circuit $I_{sc}$	9.05 A
Peak output current $I_{max}$	8.64 A

Boost converter	
Inductance *L*	15 mH
Capacitance *C*	4700 $\upmu$ F
Resistance *R*	1682 $\Omega$
Operating frequency $F_{sw}$	5 kHz
Voltage at input $V_{in}$	227.5 V
Voltage at output $V_{out}$	600 V

### Irradiance change

**Fig. 11:**
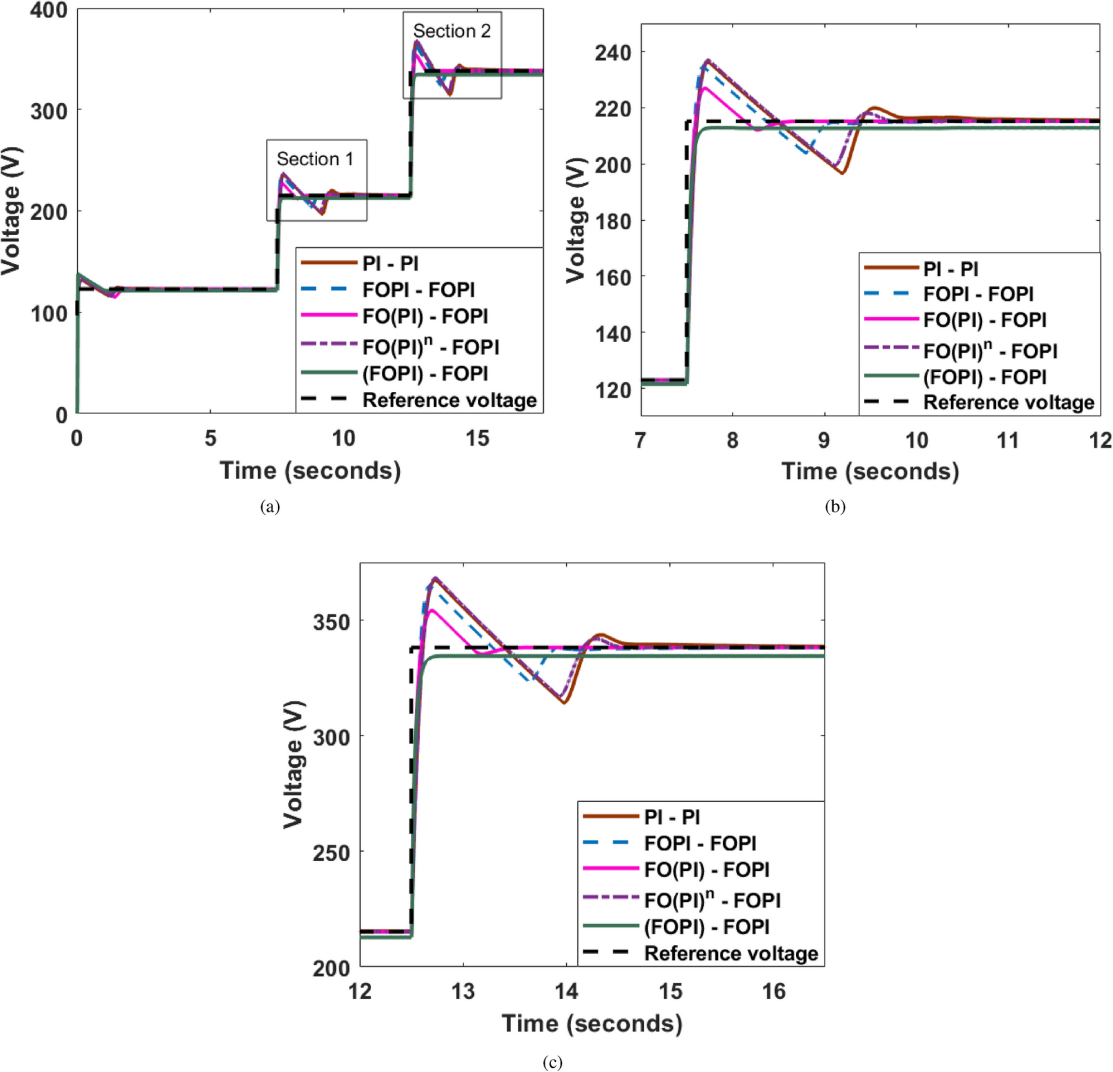
(**a**) Variation in Output voltage for different irradiation with EHO tuned controllers (**b**) Section 1-Magnified view and (**c**) Section 2-Magnified view.

A comparison of the boost converter output voltage across four types of C-FOCs tuned with EHO is depicted in Fig. 11. The simulation has been performed under different irradiation conditions of $200\ {\text{W}}/{\text{m}}^{2}$, $350\ {\text{W}}/{\text{m}}^{2}$, and $550\ {\text{W}}/{\text{m}}^{2}$ at 0, 5, and 7.5 s, respectively.

Figure 11 indicates that, although the (FOPI)-FOPI controller gives less overshoot similar to the time response graph, the steady-state error is 1.3% under Section 1 and 1.8% under Section 2 due to its overdamped response. This shows that the steady-state error increases for higher irradiance, which is unacceptable. Concerning the overshoot in the FO(PI)-FOPI controller, as irradiance increases, the overshoot percentage is 5.8% in Section 1 and 2.7% in Section 2. This is an acceptable range in terms of the percentage of overshoot. Thus, comparatively, the FO(PI)-FOPI controller gives better performance than the other cascade controllers.

### High resistive load change

**Fig. 12:**
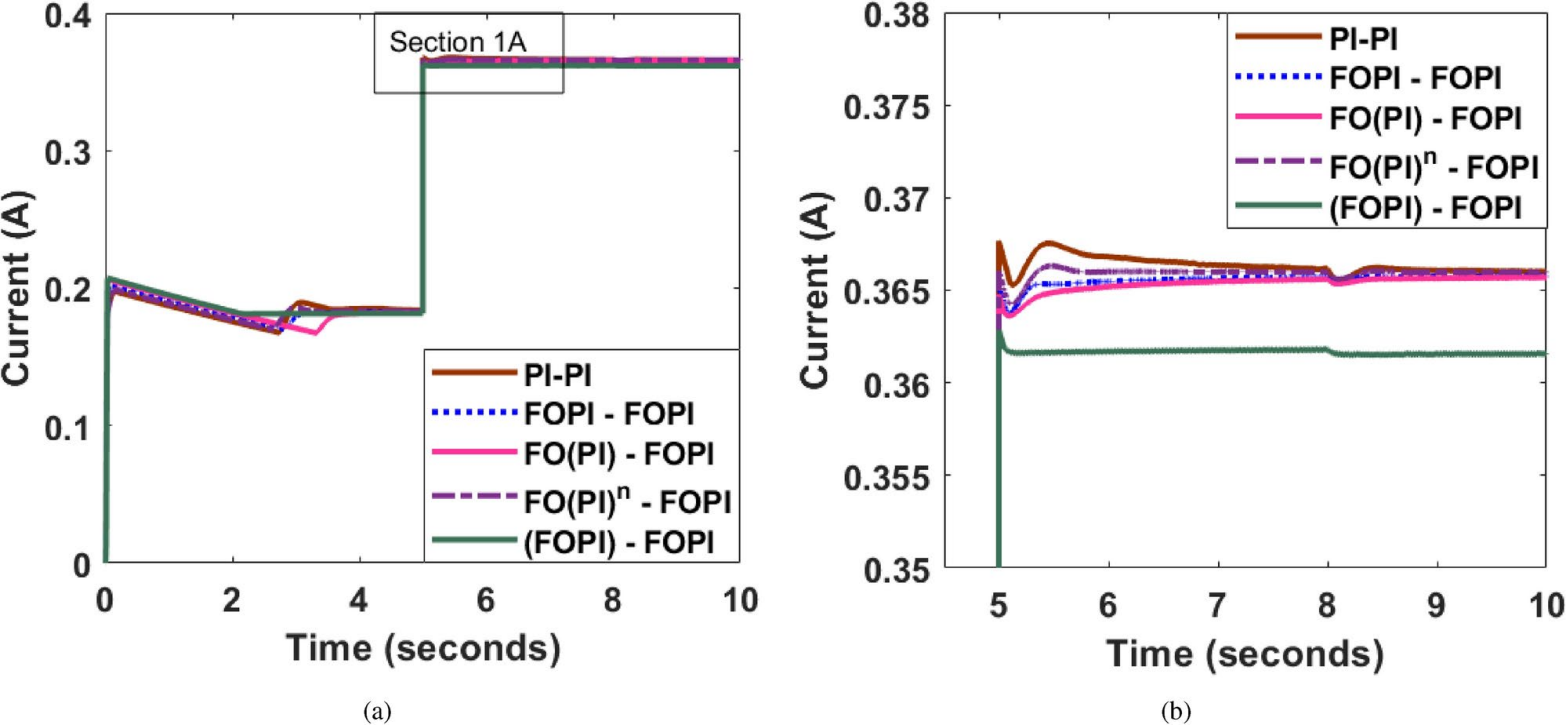
(**a**) Variation in load current for 100% dynamic load change with EHO tuned controllers (**b**) Section 1A-Magnified view.

The variation in load current under an additional 100% load change at *t* = 2.5 s has been depicted in Fig. 12. Analysis of the figure suggests that the FO(PI)-FOPI controller comparatively gives superior performance with lesser overshoot and undershoot than the other controllers.

### Random load change

**Fig. 13:**
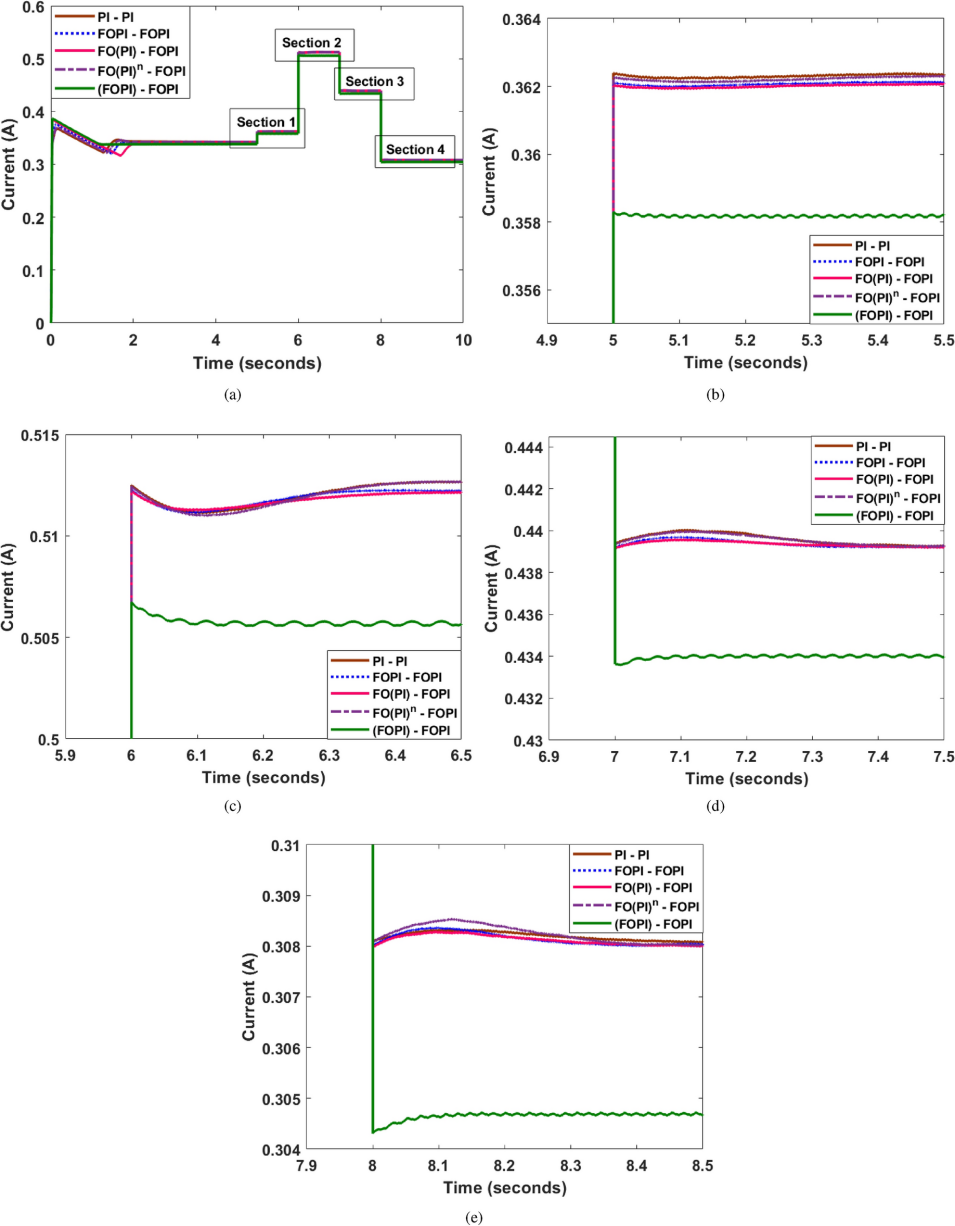
(**a**) Variation in load current for random load change (**b**) Section 1-Magnified view (**c**) Section 2-Magnified view (**d**) Section 3-Magnified view (**e**) Section 4-Magnified view.

A random dynamic load shift has been made at *t* = 5, 6, 7 and 8 s, and Fig. 13 shows the associated load current. Analyzing the figure indicates that, in comparison to the other controllers, the FO(PI)-FOPI controller performs better with minimal overshoot and undershoot.

### Inductance disparity

**Fig. 14:**
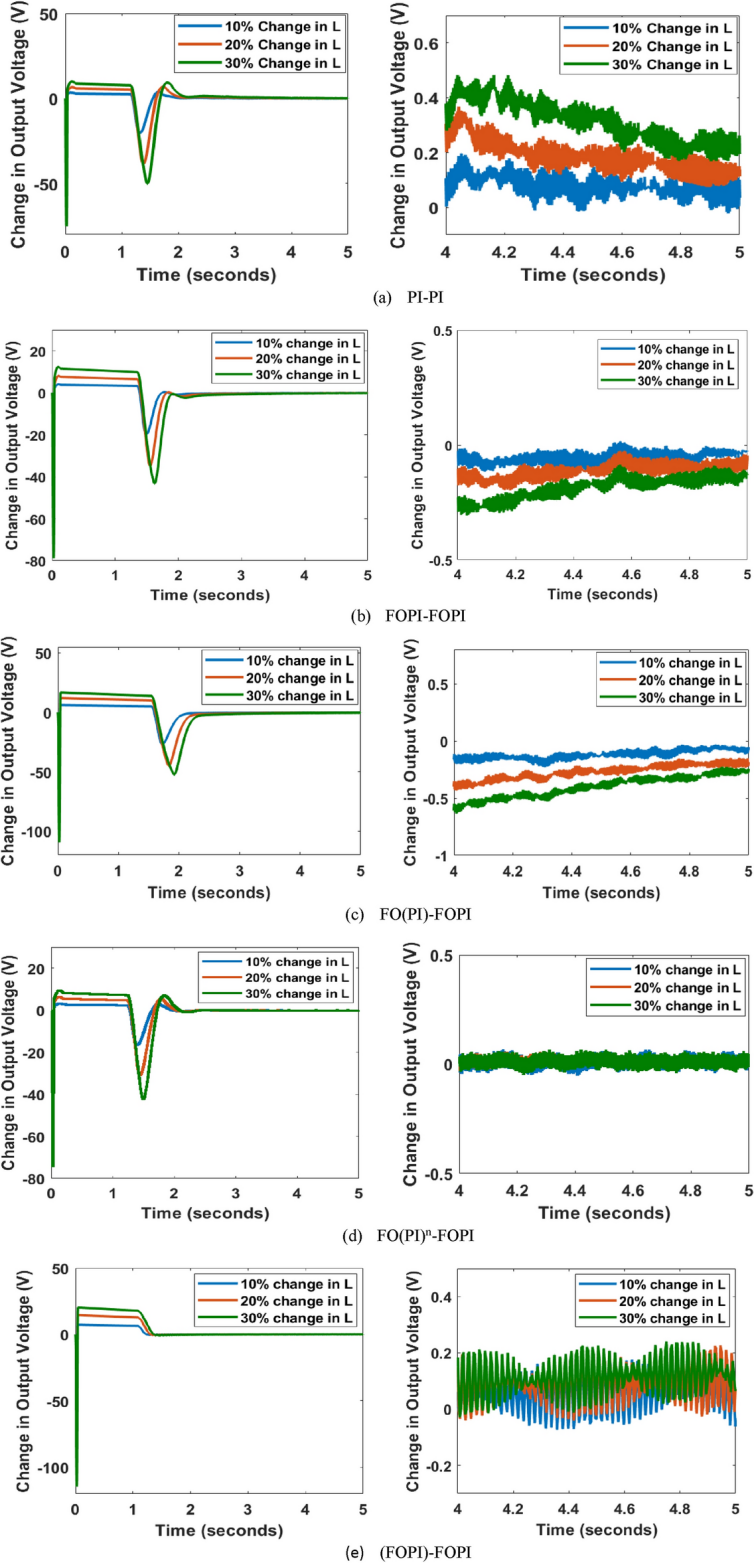
Output voltage variance as a result of inductance disparity with EHO tuned controllers with the right side graph showing the magnified view of period t = (4–5) s.

The simulation results captured on the output voltage variation with the change in L up to 30 % from the nominal value are presented in Fig. 14. The initial voltage dip contributes to the energy storage elements of the boost converter, which produces the time delay. The results can be inferred as shown below

- The variation of the voltage at the output for PI-PI controller is as far as 0.3 V as the L drifts up to 30% when observed at the steady-state time period of *t* = $( 4.8 - 5 )$ s.
- In the FOPI-FOPI controller, the output voltage varies until 0.2 V as with 30% change in L during the steady-state period *t* = $( 4.8 - 5 )$ s.
- The FO(PI)-FOPI makes the output voltage slide up to 0.25 V for the disparity of 30% in the L during the same period of *t* = $( 4.8 - 5 )$
- The disparity in L has no significant effect on the FO(PI$$)^n$$-FOPI controller during the same steady-state period.
- The output voltage deviation is 0.1 V in the case of (FOPI)-FOPI controller on a 30% change in L value for the same steady state period.

On analyzing the results, the variations for the error in response are considerably small for all the controllers, with a maximum variation of 0.3 V by the PI-PI controller. Among all the controllers, the FO(PI$$)^n$$-FOPI controller provides superior support for L disparity.

### Series inductance resistance, $r_L$ disparity

**Fig. 15:**
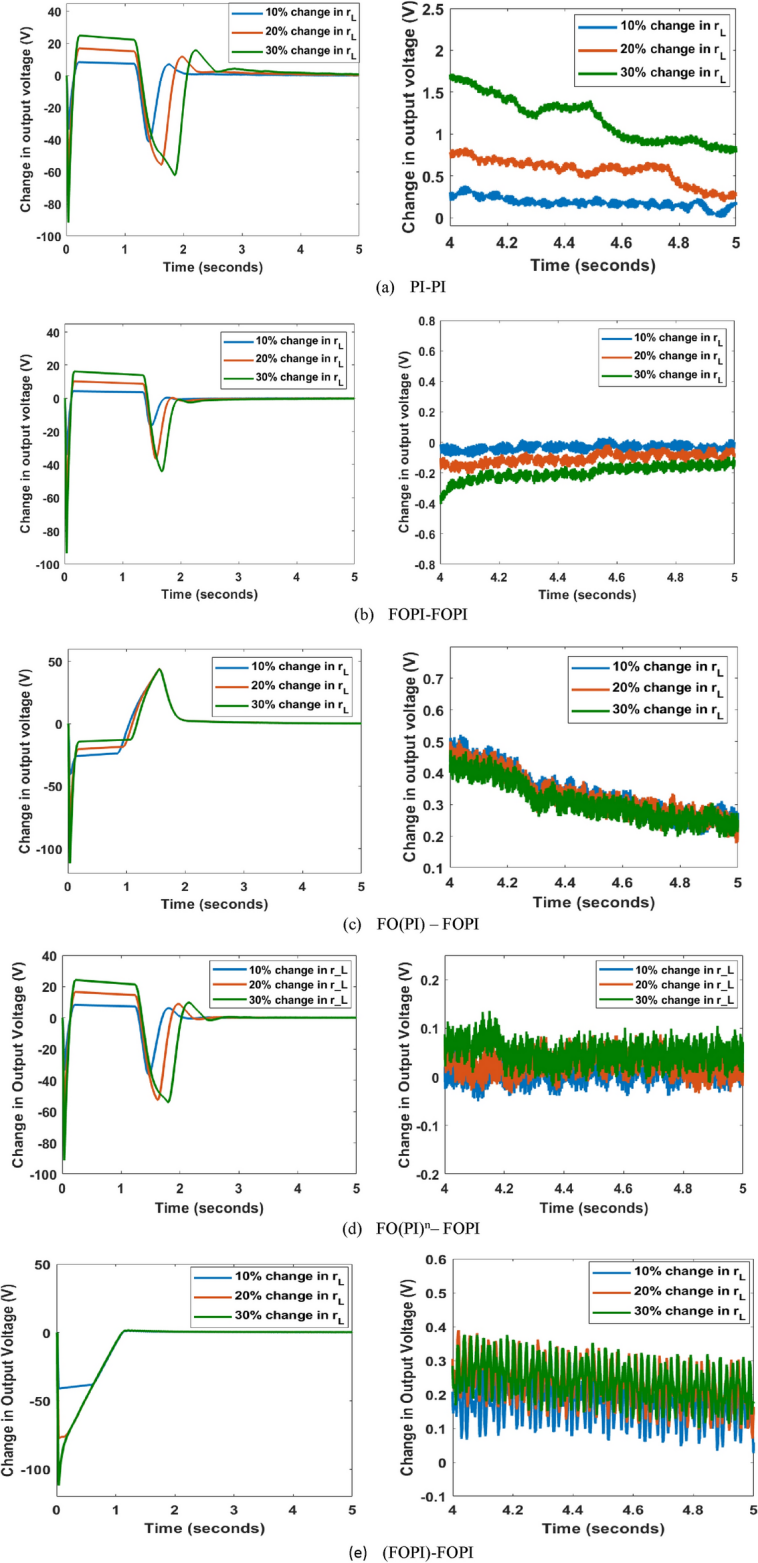
Output voltage variance as a result of Series inductance disparity with EHO tuned controllers with the right side graph showing the magnified view of period t = (4–5) s.

Figure 15 depicts the output voltage variation for $r_L$ disparity of up to 30 %. The results are discussed below

- With PI-PI controller, the shift in output voltage is 1 V for the drift in $r_L$ up to 30 % during *t* = $( 4.8 - 5 )$ s.
- FOPI-FOPI controller makes the deviation in output voltage up to 0.2 V for change in $r_L$ of 30 % at *t* = $( 4.8 - 5 )$ s.
- The variation in output voltage in response to variation of 30 % in $r_L$ is 0.25 V at *t* = $( 4.8 - 5 )$ s for FO(PI)-FOPI controller.
- The variation in output voltage with FO(PI$$)^n$$-FOPI for the same time period and $$r_L$$ value is as minimum as 0.05 V.
- The (FOPI)-FOPI controller oscillates more than the other controllers for the same value of $r_L$ and time period. The change in output voltage is, on average, 0.2 V.

In summary, all the C-FOPIs respond better than the PI-PI controller, with a substantially lower variation of output voltage with a maximum of 0.25 V.

**Fig. 16:**
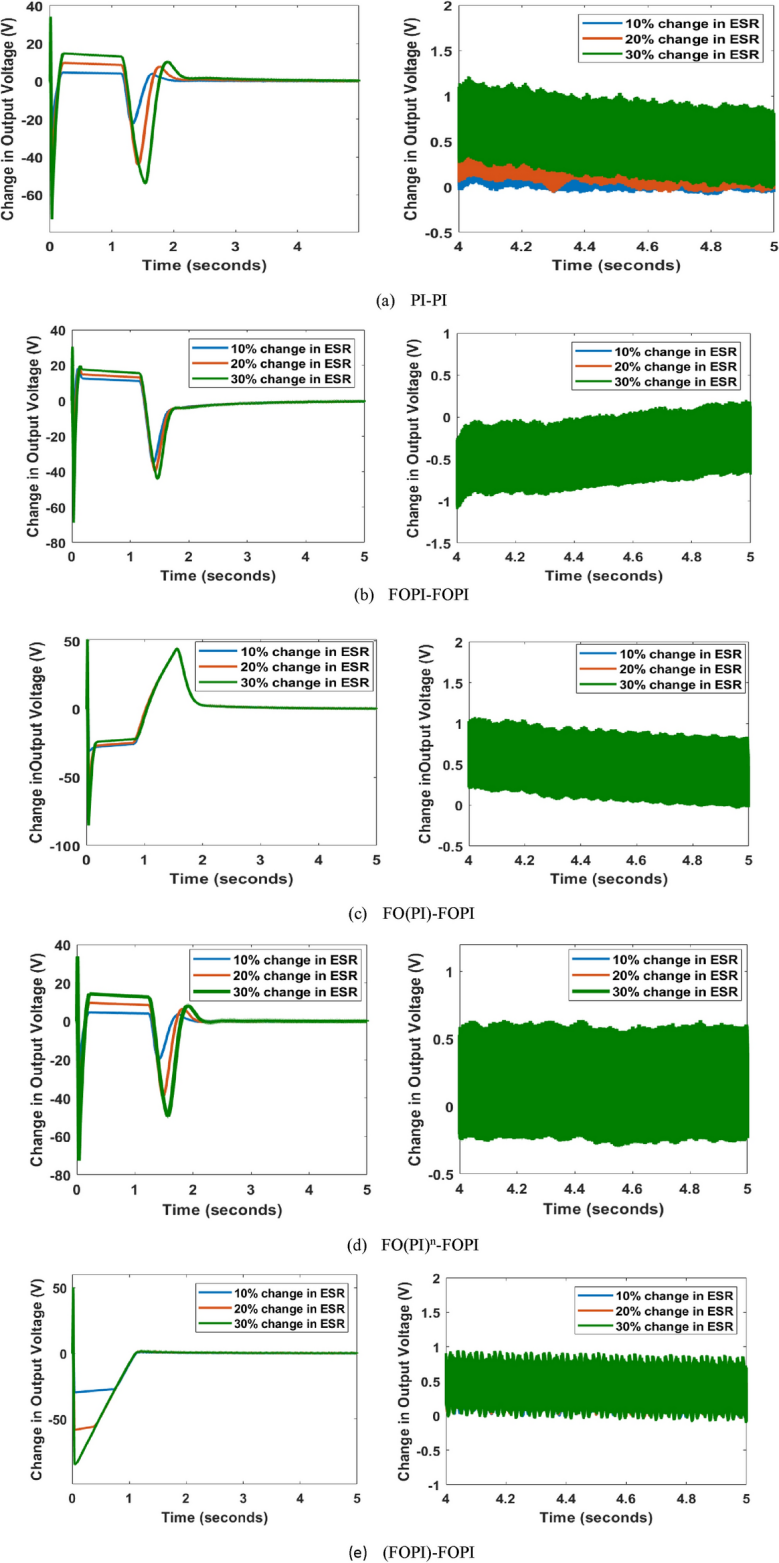
Output voltage variance as a result of ESR disparity with EHO tuned controllers, with the right side graph showing the magnified view of period t = (4–5) s.

### ESR, $r_C$ disparity

The error in response to output voltage concerning modification up to 30 % in $r_C$ is illustrated in Fig. 16. Below is a discussion of the results

- The oscillation in the error of output voltage with the PI-PI controller is around 0.9 V for deviation in $r_C$.
- The error with the FOPI-FOPI controller oscillates as far as 0.85 V for drift in $r_C$.
- With the FO(PI)-FOPI controller, the oscillation is almost 0.75 V.
- The FO(PI$$)^n$$-FOPI controller suppresses the oscillations till 0.8 V.
- The (FOPI)-FOPI controller subdues the oscillation down to 0.9 V.

In brief, the disparity in output voltage response is almost the same for changes in $r_C$ for all the controllers. When comparing the responses of all the C-FOPIs to the PI-PI controller, the former delivers exceptional responses with a minimum fluctuation of 0.75 V by the FO(PI)-FOPI controller with variations in $r_C$. This is primarily due to the wider frequency response of the FOC, which allows it to handle an expanded range of disturbances and discrepancies in converter operating conditions.

**Fig. 17:**
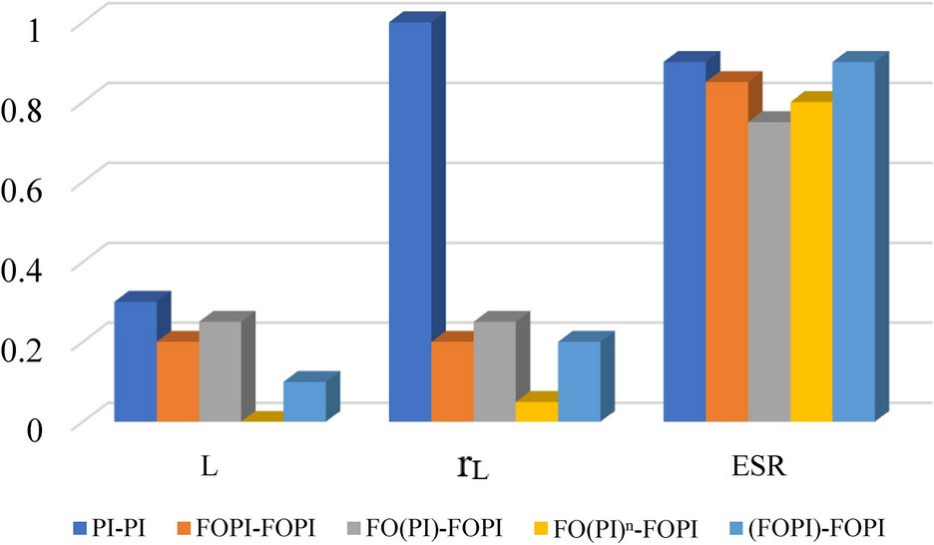
Variation in Output voltage due to parametric variation.

Although the change is relatively small, the FO(PI$$)^n$$-FOPI controller affords an improved adaptation for parametric fluctuations in L and $$r_L$$, whereas the FO(PI)-FOPI controller gives an enhanced reaction with parameter changes in $$r_C$$ among the C-FOPIs as depicted in Fig. 17.

### Efficiency analysis

**Fig. 18:**
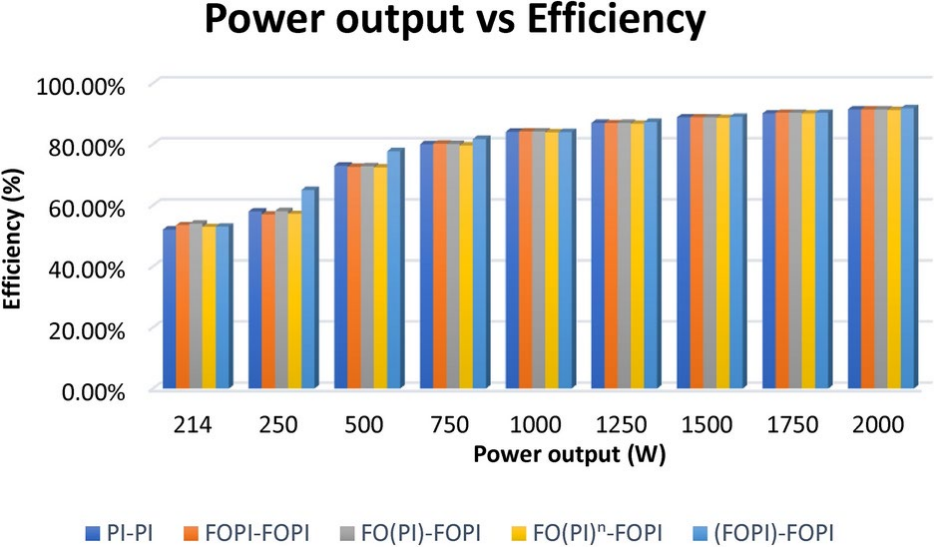
Efficiency analysis.

Power output and efficiency graph have been provided in Fig. 18 to assess how various controllers affect system efficiency. The findings show that although controllers affect accuracy in the steady state and transient response, they have little impact on overall efficiency. Across various control schemes, efficiency stays relatively consistent, indicating that significant gains occur in dynamic performance rather than energy conversion efficiency.

## Conclusion

The implementation and comparison of various forms of C-FOCs represent the principal objective of this research. Through a comparison of the various structures of FOCs, the FO(PI)-FOPI controller proves to give a greater response in terms of changing irradiance, high resistive load change, and $$r_C$$ disparity. It gives a 36.5% reduction in settling time and 50% improvement in steady-state error in comparison with the PI-PI controller. The FO(PI$$)^n$$-FOPI controller gives a better response to changes in parameters such as L and $$r_L$$ disparity, reducing the voltage variation by up to 75% compared to conventional methods. Due to their broad frequency response range, FOCs may be sensitive to high-frequency disturbances even though they are often robust. This might indicate the need for more filtering techniques, which may escalate the hardware or the processing demands substantially. The proposed controller, which is validated through simulations, has provided promising results. To confirm its performance amid realistic circumstances, an experimental validation on a physical microgrid system is still required. Considering that FOCs must be realised with an approximation transfer function of higher order to correspond to their frequency response within the region of relevance of the recursive method^77^, their practical implementation is hindered. It is frequently observed that the analog realization associated with these high-order systems becomes susceptible to distinct component endurance effects. Therefore, it ought to implement a discrete variant of the approximated transfer function of integer order. The future work will be prioritized on extending the application of FOCs in multi-converter systems, where managing the interaction between multiple converters is crucial and validating experimentally in practical setups. The application of non-linear modeling for harmonic suppression will also be considered in the subsequent research. To further confirm the suggested method’s efficacy in dynamic and unpredictable contexts, future research will also compare it to adaptive control, model predictive control (MPC), and H-infinity robust control.

## Supplementary Information

Supplementary Information.

## Data Availability

The datasets used and/or analysed during the current study are available from the corresponding author upon reasonable request.

## List of symbols

$$V_{ref}$$: Reference voltage
$V_{out}$: Boost converter output voltage
$$C_V$$: Voltage controller
$$I_{ref}$$: Current reference
$i_L$: Current through inductor
$$C_C$$: Current controller
d: Duty ratio
$K_p$, $K_i$: Proportional gain, integral gain
$$\delta$$ , *n*: Fractional orders
$K_{pc}$, $K_{pc}$, $\delta _c$: Proportional gain, integral gain, and fractional order for current controller
$K_{pv}$, $K_{pv}$, $\delta _c$: Proportional gain, integral gain, and fractional order for voltage controller
$r_L$: Series inductance resistance
$r_C$: ESR of the output capacitor
