# Supplementary material for: A relative analysis to cascaded fractional-order controllers in microgrid non-minimum phase converters using EHO
Source: Sci Rep. 2025 Mar 25;15:10333. doi: 10.1038/s41598-025-94690-y (PMC11937251; doi:10.1038/s41598-025-94690-y)
Supplement: Supplementary file 1 — Supplementary Information. [file 41598_2025_94690_MOESM1_ESM.pdf]

Appendix

Derivation of transfer functions from state equations

From the state equations, the loop transfer functions of voltage and current are derived[1] and detailed as follows:

$$\begin{bmatrix} \frac{di_L(t)}{dt} \\ \frac{dv_{out}(t)}{dt} \end{bmatrix} = \underbrace{\begin{bmatrix} 0 & -\frac{(1-d)}{L} \\ \frac{1-d}{C} & -\frac{1}{RC} \end{bmatrix}}_{A} \underbrace{\begin{bmatrix} i_L \\ v_{out} \end{bmatrix}}_{x} + \underbrace{\begin{bmatrix} \frac{v_{out}}{L} \\ -\frac{i_L}{C} \end{bmatrix}}_{B_1} d + \underbrace{\begin{bmatrix} \frac{1}{L} \\ 0 \end{bmatrix}}_{B_2} v_{in} \tag{1}$$

$$i_L = \underbrace{\begin{bmatrix} 1 & 0 \end{bmatrix}}_{C_1} \begin{bmatrix} i_L \\ v_{out} \end{bmatrix} \tag{2}$$

$$v_{out} = \underbrace{\begin{bmatrix} 0 & 1 \end{bmatrix}}_{C_2} \begin{bmatrix} i_L \\ v_{out} \end{bmatrix} \tag{3}$$

From the state equations (1), (2) and (3), $G_c(s)$ and $G_o(s)$ which is the transfer function in the open loop, have been obtained as

$$G_c(s) = \frac{i_L(s)}{d(s)} = C_1(sI - A)^{-1}B1 \tag{4}$$

On replacing the coefficients of A, B_1 and C_1, the equation (4) can be obtained as

$$G_c(s) = \begin{bmatrix} 1 & 0 \end{bmatrix} \left(\begin{bmatrix} s & 0 \\ 0 & s \end{bmatrix} - \begin{bmatrix} 0 & -\frac{(1-d)}{L} \\ \frac{(1-d)}{C} & -\frac{1}{RC} \end{bmatrix} \right)^{-1} \begin{bmatrix} \frac{v_{out}}{L} \\ -\frac{1}{C} \end{bmatrix} \tag{5}$$

$G_c(s)$ can thus be obtained by rewriting equation (5) as shown below

$$G_c(s) = \frac{i_L(s)}{d(s)} = \frac{2I_L(1-d) + V_{out}C}{LCs^2 + \frac{L}{R}s + (1-d)^2} \tag{6}$$

The voltage transfer function at the outer loop, $G_v(s) = \frac{v_{out}(s)}{i_L(s)}$ is derived as

$$G_v(s) = \frac{G_o(s)}{G_c(s)} = \frac{\frac{v_{out}(s)}{d(s)}}{\frac{i_L(s)}{d(s)}} = \frac{v_{out}(s)}{i_L(s)} \tag{7}$$

$$G_o(s) = \frac{v_{out}(s)}{d(s)} = C_2(sI - A)^{-1}B_1 \tag{8}$$

On substituting the coefficients of A, B_1 and C_2 in equation (8), the following equation can be attained

$$G_o(s) = \begin{bmatrix} 0 & 1 \end{bmatrix} \left(\begin{bmatrix} s & 0 \\ 0 & s \end{bmatrix} - \begin{bmatrix} 0 & -\frac{(1-d)}{L} \\ \frac{(1-d)}{C} & -\frac{1}{RC} \end{bmatrix} \right)^{-1} \begin{bmatrix} \frac{v_{out}}{L} \\ -\frac{i_L}{C} \end{bmatrix} \tag{9}$$

On rewriting equation (9), $G_o(s)$ can be derived as follows

$$G_o(s) = \frac{v_{out}(s)}{d(s)} = \frac{-LI_Ls + V_{out}(1-d)}{LCs^2 + \frac{L}{R}s + (1-d)^2} \tag{10}$$

Thus, with respect to equation (7) $G_v(s)$ can be derived as shown below

$$G_v(s) = \frac{G_o(s)}{G_c(s)} = \frac{v_{out}(s)}{i_L(s)} = \frac{-LI_Ls + V_{out}(1-d)}{V_{out}Cs + 2I_L(1-d)} \tag{11}$$

References

1. Smitha, K., Aryar, P. G., Bijlwan, R. & Angadi, S. Steady state analysis of pid controlled boost converter using state space averaging technique. In *National Conference-NCPE-2k15, organized by KLE Society's Dr. MS Sheshgiri College of Engineering & Technology, Belagavi, Special issue published by Multidisciplinary Journal of Research in Engineering and Technology, Pg*, 100–110 (2012).
